# Transformation of *Fonsecaea pedrosoi* into sclerotic cells links to the refractoriness of experimental chromoblastomycosis in BALB/c mice via a mechanism involving a chitin-induced impairment of IFN-γ production

**DOI:** 10.1371/journal.pntd.0006237

**Published:** 2018-02-26

**Authors:** Bilin Dong, Zhongsheng Tong, Ruoyu Li, Sharon C.-A. Chen, Weihuang Liu, Wei Liu, Yao Chen, Xu Zhang, Yiqun Duan, Dongsheng Li, Liuqing Chen

**Affiliations:** 1 Center for Infectious Skin Diseases, Department of Dermatology, No. 1 Hospital of Wuhan, Wuhan, China; 2 Research Center for Medical Mycology, Peking University, Beijing, China; 3 Center for Infectious Diseases and Microbiology, Laboratory Services, ICPMR, Westmead Hospital, University of Sydney, Westmead, Australia; 4 Medical Research Center of Wuhan University, Wuhan, China; 5 Institute of Hydrobiology, Chinese Academy of Sciences, Wuhan, China; Universidad de Antioquia, COLOMBIA

## Abstract

*Fonsecaea pedrosoi* (*F*. *pedrosoi*) is the most common agent of chromoblastomycosis. Transformation of this fungus from its saprophytic phase into pathogenic sclerotic cells in tissue is an essential link to the refractoriness of this infection. Experimental studies in murine models have shown that the absence of CD4+ T cells impairs host defense against *F*. *pedrosoi* infection. Clinical research has also suggested that a relatively low level of the Th1 cytokine INF-γ and inefficient T cell proliferation are simultaneously present in patients with severe chromoblastomycosis upon in vitro stimulation with ChromoAg, an antigen prepared from *F*. *pedrosoi*. In the present study, we show that in mice intraperitoneally infected with *F*. *pedrosoi*-spores, -hyphae or in vitro-induced sclerotic cells respectively, the transformation of this causative agent into sclerotic cells contributes to a compromised Th1 cytokine production in the earlier stage of infection with impaired generation of neutrophil reactive oxygen species (ROS) and pan-inhibition of Th1/Th2/Th17 cytokine production with disseminated infection in the later stage by using a CBA murine Th1/Th2/Th17 cytokine kit. In addition, we have further demonstrated that intraperitoneal administration of recombinant mouse IFN-γ (rmIFN-γ) effectively reduces the fungal load in the infected mouse spleen, and dampens the peritoneal dissemination of *F*. *pedrosoi*-sclerotic cells. Meanwhile, exogeneous rmIFN-γ contributes to the formation and maintenance of micro-abscess and restores the decrease in neutrophil ROS generation in the mouse spleen infected with *F*. *pedrosoi*-sclerotic cells. Of note, we have once again demonstrated that it is a chitin-like component, but not β-glucans or mannose moiety, that exclusively accumulates on the outer cell wall of *F*. *pedrosoi*-sclerotic cells which were induced in vitro or isolated from the spleens of intraperitoneally infected BALB/c mice. In addition, our results indicate that decreased accumulation of chitin on the surface of live *F*. *pedrosoi*-sclerotic cells after chitinase treatment can be self-compensated in a time-dependent manner. Importantly, we have for the first time demonstrated that exclusive accumulation of chitin on the transformed sclerotic cells of *F*. *pedrosoi* is involved in an impaired murine Th1 cytokine profile, therefore promoting the refractoriness of experimental murine chromoblastomycosis.

## Author summary

Chromoblastomycosis is a chronic, often debilitating, suppurative, granulomatous mycosis of the skin and subcutaneous tissues beginning after traumatic implantation of melanized fungi, of which *Fonsecaea pedrosoi* (*F*. *pedrosoi*) is considered the most common agent. Data suggest that the morphological transformation to the sclerotic cell form is linked to impaired clearance of the fungus in infected tissue. Notably, previous studies showed that the CD4+ T lymphocytes play an active role in host defense against *F*. *pedrosoi* in murine chromoblastomycosis and that a relatively low level of Th1 cytokine INF-γ and inefficient T cell proliferation are present in patients with the severe form of chromoblastomycosis. In the present work, by studying the BALB/c mice intraperitoneally infected with *F*. *pedrosoi*-spores, -hyphae or in vitro-induced sclerotic cells respectively, we demonstrate that the transformation of this agent into sclerotic cells contributes to a compromised Th1 cytokine production in the earlier stage of infection with impaired neutrophil ROS generation and pan-inhibition of Th1/Th2/Th17 cytokine production with disseminated infection in the later stage. In addition, we have further demonstrated that intraperitoneal administration of exogeneous IFN-γ greatly reduces the fungal load in the spleens of BALB/c mice infected with *F*. *pedrosoi*-sclerotic cells and inhibits the peritoneal dissemination of this agent. Meanwhile, exogeneous IFN-γ contributes to the formation and maintenance of micro-abscess and restores the decrease in neutrophil ROS generation in the nidus. Importantly, we have for the first time demonstrated that exclusive accumulation of chitin on the outer cell wall of *F*. *pedrosoi*-sclerotic cells, but not the -spores or -hyphae, contributes to an impaired murine Th1 cytokine production upon in vivo stimulation with this agent.

## Introduction

Chromoblastomycosis is a chronic granulomatous mycosis of the skin and subcutaneous tissues caused by melanized fungi, of which *Fonsecaea pedrosoi* (*F*. *pedrosoi*) is considered the most common agent [1–3]. Although systemic invasion is rare, localized chromoblastomycosis may be progressive, often debilitating, and is associated with clinical complications including lymphoedema and malignant transformation of such long-standing lesions [4–7]. The effects of currently used antifungal therapies for this disease have thus far proven to be poor [3].

In our previous study, we demonstrated that T lymphocytes play an active role in defense against *F*. *pedrosoi* infection by using BALB/c and athymic BALB/c murine models of experimental chromoblastomycosis [8]. It is known that T helper (Th) cells regulate host immune responses against fungi through the secretion of distinct cytokine profiles [9]. IL-17 secreted by Th-17 cells mobilizes neutrophils required for anti-fungal responses [10–12], whereas Th1-produced IFN-γ optimally activates neutrophils and subsequent phagocytosis of fungi [9]. Specifically, absence of CD4+ T cells impairs host defense against *F*. *pedrosoi* infection in mice [13]. Of note, it has been documented that predominant production of INF-γ, low levels of IL-10 and efficient T cell proliferation were induced by ChromoAg, an antigen prepared from *F*. *pedrosoi*, in patients with a mild form of chromoblastomycosis due to *F*. *pedrosoi* [14, 15]. By contrast, in patients with severe form of the disease, predominant production of IL-10, low level of IFN-γ and inefficient T cell proliferation were observed [14, 15]. What’s more, IFN-γ production in patients with chromoblastomycosis due to *F*. *pedrosoi* after 12 months of oral antifungal treatment decreased significantly upon in-vitro stimulation with ChromoAg when compared with that in these patients after 6 months of treatment [16]. Considering that the Th1 cytokine pattern could lead to the development of cellular immunoprotective response, some scholars suggested that the Th1 hypo-responsiveness to *F*. *pedrosoi* antigens in patients with chromoblastomycosis may help explain the high relapse of this disease, although it can be partially or transitionally restored with conventional treatments. [9, 14, 16]. Therefore, we infer that the long-term host-fungus interaction will impair the original cellular immune response of the host although the agents of chromoblastomycosis including *F*. *pedrosoi* usually invade individuals with fully functional immunity by traumatic inoculation [17].

Characteristically, when embedded in tissue, most etiological agents of chromoblastomycosis including *F*. *pedrosoi* will transform into the parasitic form, i.e. the sclerotic cell form [17–20]. It is evidenced that this morphological change can enhance the ability of parasitic *F*. *pedrosoi* to defend against host elimination, which is linked to the refractoriness of chromoblastomycosis [17, 20]. Briefly, the characteristics of sclerotic cells facilitating their immune escape basically include optimized surface/volume ratio favoring increased melanin deposition and higher ectophosphatase activity when compared with those of saprophytic mycelia or conidia [17, 20, 21]. However, it remains to be elucidated whether the in-vivo transformation of *F*. *pedrosoi* into sclerotic cells in infected tissue has an impact on host immune responses including Th1/Th2/Th17 cells development.

Of note, we have further demonstrated that it is a chitin-like component, rather than β-glucan or mannose moiety, that exclusively accumulates on the outer cell wall of in vitro-induced sclerotic cells of *F*. *pedrosoi* in our previous study [8]. Chitin, a robust β-1, 4-linked homopolymer of N-acetylglucosamine (GlcNAc), is crosslinked with β-glucan and glycoprotein to form a complex network, which maintains the overall integrity of fungal cell wall [22–26]. Although purified chitin is a plain polysaccharide that, at the nano level, presents itself as a highly associated structure [27], chemical linkages between fungal chitin and β-glucans may change with cell wall growth and remodeling [28, 29]. Until now, inflammatory cytokine patterns and Th cell development induced by natural chitin on fungal cell wall remain largely unknown. Notably, a recent study has demonstrated that *Candida albicans* chitin can induce polarization of human macrophage towards M2 phenotype with increased arginase-1 activity [30]. It is well established that the anti-inflammatory cytokines secreted by human or mice M2 macrophages contribute to the impairment of Th1 cell development [31, 32]. Therefore, we hypothesize that chitin accumulation on the outer cell wall of *F*. *pedrosoi*-sclerotic cells may change the original Th1/Th2/Th17 cytokine patterns in host and contributes to an inhibited IFN-γ production during the long-term host-fungus interaction.

In contrast with the progressive healing pattern presented in the infected footpads of BALB/c mice which were subcutaneously inoculated with *F*. *pedrosoi* [8, 33], the recalcitrant infection in immunocompetent mice established by intraperitoneal injection of this agent can well reflect the chronicity of human chromoblastomycosis [8]. Accordingly, we hope to establish a chronic chromoblastomycosis model in mice by intraperitoneal injection with *F*. *pedrosoi*-spores, -hyphae and sclerotic cells respectively in the present study and aim to verify our hypothesis that the transformation of this etiological agent into sclerotic cell form can result in a compromised IFN-γ production via a mechanism involving chitin accumulation on the surface of sclerotic cells, and therefore promotes the refractoriness of experimental murine chromoblastomycosis.

## Materials and methods

### Source of mice

Immunocompetent and athymic (nu/nu) BALB/c male mice (SPF, 5–6 wk old) were purchased from the Animal Laboratory Center, Wuhan University, and maintained in special pathogen-free conditions.

### Ethical statement

All animal experiments in this study were performed in accordance with the recommendations in the Guide for the Care and Use of Laboratory Animals of National Institutes of Health. Our study protocol was approved by the Institutional Animal Care and Use Committee of No.1 Hospital of Wuhan (project license number: WHB201511012). To minimize suffering, mice were anaesthetized prior to sacrifice.

### Fungal strain and preparation of saprophytic cultures

*F*. *pedrosoi* strain (WH10-002) (GenBank no: GQ420654.1) was obtained from the patient with long-standing chromoblastomycosis and identified by ITS sequencing in combination with morphological analysis. The strain was cultivated on Potato Dextrose Agar (PDA) (Difco, BD) supplemented with chloramphenicol at 50 μg/ml at 28°C and was periodically transferred at 60-day intervals for preservation.

To prepare the *F*. *pedrosoi*-spores, the stock culture was inoculated onto the Oatmeal Agar (Difco, BD) and cultured for 2 weeks at 28°C. The spores were obtained by the addition of normal saline (NS) into the agar and scraping of the reproductive mycelium with a disposable sterilized swab. Afterwards, the saline solution was collected and immediately filtered twice through the four-layer medical sterilized gauze. Finally, the filtrate containing purified spores was washed twice in NS by centrifugation at 4000 rpm for 5 min and was adjusted to a final concentration of 5.0×10^8^/mL in NS solution with a Neubauer chamber.

To prepare *F*. *pedrosoi*-hyphae, the stock culture was inoculated into the Sabouraud Dextrose Broth (SDB) (Difco, BD), and cultured for 2 weeks at 28°C. Afterwards, the mycelial masses in broth were unfolded with a glass homogenizer by pushing and pulling the plunger gently for several times, and then the homogenous suspension was filtered through a nylon filter (200 mesh), where the remnant mycelium masses were retained. The filtrates containing solitary, short hyphal fragments were further washed twice in NS by centrifugation at 4000 rpm for 5 min and adjusted to a final concentration of 5.0×10^8^/mL with a Neubauer chamber. The separation between the septa was used to distinguish individual hyphal cells, which were then counted.

### In vitro induction of *F*. *pedrosoi*-sclerotic cells

To induce the formation of sclerotic cells, the 15-day old *F*. *pedrosoi*-hyphae grown in SDB were re-inoculated into the synthetic basal medium (ATCC medium 830), pH 5.0, with the following composition (g/L): MgSO_4_, 0.1; NH_4_NO_3_, 1.5; KH_2_PO_4_, 1.8; Biotin, 5×10^−5^; thiamine-HCl, 1.0×10^−4^; Glycerol, 6.5, as previously described [8, 34]. In addition, platelet-activating factor was added at a final concentration of 10^−6^ M. After incubation at 36°C for 50 days, the formation of sclerotic cells was confirmed by microscope examination.

For morphological analysis and intraperitoneal infection of mice, the homogeneous suspension mainly composed of sclerotic cells was prepared as described in the preparation of *F*. *pedrosoi*-hyphae, and was adjusted to a final concentration of 5.0×10^8^/mL in NS solution with a Neubauer chamber.

### The viability test for fungal cells

FUN1 Cell Stain (Cat: F7030, Invitrogen), a unique two-color fluorescent viability probe for yeast and fungi, was introduced to test the viability of *F*. *pedrosoi*-spores, -hyphae and in vitro-induced sclerotic cells. Briefly, the three types of fungal cells were pre-stained with FUN1 dye, and their viability was measured using flow cytometer (BD FACS Aria Ⅲ) according to the protocol by setting the 488 nm laser line as the excitation source, standard FITC channel for detection of green emission (dead/metabolically inactive cells) and PE channel for red emission (live/metabolically active cells). The fungal cells with red emission or two-color emissions were considered viable. In addition, the fungal cells without FUN1 treatment were set as self-fluorescence control. The fungal cells which were heat-killed by incubation in a water-bath at 70°C for 90 min and stained with FUN1 were set as the dead cell control.

### Intraperitoneal administration of exogeneous rmIFN-γ

For the group of mice (n = 24) to be injected intraperitoenally with *F*. *pedrosoi*-sclerotic cells and recombinant mouse IFN-**γ** (rmIFN-γ) (Cat: 485-MI/CF, R&D systems), each one was intraperitoneally injected with 300 μL of 0.9% normal saline containing 25 μg IFN-**γ** once a day for consecutive 5 days from 1 day before fungal inoculation and then with 12.5 μg IFN-**γ** every other day from 7 till 50 days after inoculation.

### Intraperitoneal infection with *F*. *pedrosoi*-spores, -hyphae and in vitro-induced sclerotic cells

Ten minutes prior to infection, the BALB/c mice were anaesthetized by intraperitoneal injection with 0.4 μl of Anasedan and 0.35 ml of Dopalen per kg body weight. Afterwards, the mice were divided into three groups (n = 12 for *F*. *pedrosoi*-spore or -hyphae group; n = 24 for -sclerotic cells group) and were respectively intraperitoneally injected with 500 μl of *F*. *pedrosoi*-spores, -hyphae or in vitro-induced sclerotic cells suspension at a concentration of 5.0×10^8^/mL using a 1-mL syringe with 21 Gauge x 1 Inch needle. Meanwhile, for the group of mice pre-injected with IFN-**γ** as above-mentioned, each one was intraperitoneally inoculated with 500 μl of *F*. *pedrosoi*-sclerotic cells suspension. The mice intraperitoneally injected with 500 μl of normal saline were set as inoculation control (n = 12).

For monitoring the development of intraperitoneal infection, the mice in the experimental and control groups were sacrificed, and the clinical features of the infected spleens and other abdominal organs were analyzed at 10, 30 and 50 days post-inoculation (n = 4 per group at each indicated time point). In addition, histological examination of the infected spleens was performed at the time points as indicated above, and detection of the causative agent in tissue was also included.

### Monitoring of fungal survival in the infected tissues of mice

For monitoring of fungal survival in the involved tissues, the spleens or other abdominal tissues isolated from BALB/c mice at 30 days after intraperitoneal inoculation with *F*. *pedrosoi*-spores and at 50 days after inoculation with -hyphae or induced sclerotic cells were respectively prepared and cultured on PDA plate at 28°C for at least 21 days.

### Evaluation of splenic fungal burden in mice

For the group intraperitoneally inoculated with *F*. *pedrosoi*-sclerotic cells and exogeneous rmIFN-γ and that merely with *F*. *pedrosoi*-sclerotic cells, the mice were sacrificed respectively at 10 days, 30 days and 50 days post-inoculation (n = 4 per group at each indicated time point), and the spleens were isolated asceptically. Then the spleen of each mouse was respectively homogenized with a glass homogenizer as mentioned above, and the homogenous suspension was filtered through a nylon filter (200 mesh). A total of 2 mL of filtrate from each sample was collected and 10-fold serially diluted (1−10^3^) in sterile normal saline. Afterwards, 100 μL of the original filtrate or the diluent was coated onto each PDA plate (n = 5 for each indicated dilution) at 28°Cfor 5 days. The fungal load in the infected spleen was measured by counting fungal colonies on the plate inoculated with appropriate diluents, and was represented as colony forming unit (CFU).

### Detection of neutrophil ROS in the infected mouse spleens

For measurement of neutrophil ROS, the infected nodules were aseptically isolated from the spleens of BALB/c mice respectively inoculated with *F*. *pedrosoi*-spores, -hyphae, and -sclerotic cells at 10 days post-inoculation (n = 4 per group). In addition, for mice intraperitoneally injected with *F*. *pedrosoi*-sclerotic cells and exogeneous rmIFN-γ (n = 4 at each indicated time point), neutrophil ROS in the infected spleens was measured consecutively at 10 days, 30 days and 50 days post-inoculation. Briefly, single-cell suspensions were prepared from the nodules using mechanical trituration method, and were adjusted to a final concentration of 3.0×10^5^/mL in RPMI 1640 after washing twice with PBS by centrifugation at 4000 rpm for 5 min. The neutrophils were selected by APC-conjugated anti-mouse Ly-6G (Gr-1) mAb (Cat: 17-9668-82, eBioscience) as well as side scatter (SSA) according to the protocol. The cells incubated with APC-conjugated rat-derived IgG2a kappa were set as isotype control. ROS levels in neutrophils were measured using 2’, 7’-dichlorodihydrofluorescin diacetate (DCHF-DA) (Cat: D6883, Sigma-aldrich) as fluorescence probe and were represented as mean fluorescence intensity (MFI). The neutrophils without adding DCHF-DA were set as blank control.

### Dectin-1, Dectin-2 and MR binding assay

For measurement of Dectin-1, Dectin-2 and MR binding affinity, the homogeneous suspensions were prepared from saprophytic *F*. *pedrosoi*-spores, -hyphae and in vitro-induced sclerotic cells which were killed by heat treatment in a water bath at 70°C for 90 min or not. Afterwards, the live and heat-killed fungal cells were adjusted to 2.0×10^6^ cells in 100 μl phosphate buffer saline (PBS) containing 2% bovine serum albumin (BSA) for use. After incubation for 60 min at 37°C with 10 μg/mL of murine-derived recombinant Dectin-1, Dectin-2 and Mannose Receptor (MR) (Cat: 1756-DC-050, 1525-DC-050 and 2535-MM-050, R&D systems) respectively and a wash in PBS, 100 μl of above-mentioned fungal cell suspensions were incubated with 10 μl of PE-conjugated anti-murine Dectin-1, APC-conjugated anti-murine Dectin-2 or Alexa Fluor-488-conjugated anti-murine MR (FAB17561P, FAB1525A and FAB2535G, R&D systems) correspondingly for another 45 min at 4°C. After further washing in PBS, the fungal cells were resuspended in 1 ml of ice-cold PBS. Finally, the binding affinity of murine-derived Dectin-1, Dectin-2 or MR to the surface of fungal cells was measured respectively by confocal laser scanning microscopy (LSM710, Zeiss) and flow cytometer (BD FACS Aria Ⅲ). The fungal cells incubated with PE-congugated Rat IgG2a, APC- or Alexa Fluor-488-conjugated Goat IgG were set as isotype control. The excitation-emission wavelengths of 488/525 nm were used for Fluor-488 assay, 488/585 nm for PE assay, and 633/670 nm for APC assay.

### Chitinase treatment

For treatment of live and heat-killed sclerotic cells induced in vitro, the homogeneous suspensions were prepared in a similar way as mentioned above and were adjusted to a final concentration of 5.0×10^8^/mL. Afterwards, a total of 5.0×10^8^ cells were incubated with 2 mL PBS containing 10 U chitinase (C6137, Sigma-aldrich) at 37°C overnight with shaking at 160 rpm. Finally, live and heat-killed sclerotic cells within 24 hours, at 7 days and 14 days after chitinase treatment were washed for three times with PBS and re-suspended in PBS for use.

For treatment of in vivo-transformed sclerotic cells, the infected spleens were isolated from BALB/c mice at 50 days after intraperitoneal inoculation with hyphae and were prepared as single-cell suspensions at a final concentration of 5.0×10^8^/mL. Afterwards, the cell suspensions including in vivo-transformed sclerotic cells were treated with chitinase in a similar way as described above.

### WGA binding assay

In the present study, FITC-conjugated Wheat Germ Agglutinin (WGA; Cat: L4895, Sigma-aldrich) (1 mg/mL) was used to detect chitin distribution on the cell wall of *F*. *pedrosoi* according to the manufactures’ protocol and previous studies [8, 29]. For measurement of WGA binding affinity to *F*. *pedrosoi* cultured in vitro, the homogeneous suspensions of live or heat-killed *F*. *pedrosoi*-spores, -hyphae, and induced sclerotic cells before and after chitinase treatment at indicated time points as mentioned in “Chitinase treatment” section were respectively prepared and were adjusted to a final concentration of 2.0×10^6^ cells in 100 μl of PBS containing 2% BSA. After incubation with 10 μl of FITC-conjugated WGA at 4°C for 90 min, 100 μl of above-mentioned fungal cell suspensions were washed in PBS for three times and resuspended in 1 ml of ice-cold PBS. Finally, the binding affinity of WGA to the surface of fungal cells was measured using confocal laser scanning microscopy (LSM710, Zeiss) and flow cytometer (BD FACS Aria Ⅲ). Fungal cells without any treatment were set as self-fluorescence control. The excitation-emission wavelengths of 488/525 nm were used for FITC assay. Furthermore, three-dimensional conformation of chitin distribution on the fungal cells, which was represented by FITC-conjugated WGA, was reconstructed according to confocal tomoscanning. Each cross-section thickness was set as 0.5 μm.

For measurement of WGA binding affinity to in vivo-transformed *F*. *pedrosoi*-sclerotic cells, the single-cell suspensions were prepared from the infected spleens of BALB/c mice at 50 days after intraperitoneal inoculation with hyphae and were adjusted to a final concentration of 2.0×10^6^ cells in 100 μl of PBS containing 2% BSA as mentioned above. Afterwards, the binding properties of FITC-conjugated WGA to the in vivo-transformed sclerotic cells in the suspensions before and within 24 hours after chitinase treatment were respectively measured using confocal laser scanning microscopy (LSM710, Zeiss) in a similar way as described above.

### Detection of endogeneous chitinase via immunohistochemistry and western blotting

For detection of chitinase using immunohistochemistry (IHC), paraffin-embedded tissue sections were prepared from the *F*. *pedrosoi*-infected footpads of nu/nu-BALB/c mice and spleens of BALB/c mice respectively at 80 days and 50 days post-inoculation. IHC staining was performed as previously described [35]. Briefly, the expression and localization of endogenous chitinase in the infected tissues were detected using rabbit-derived Anti-AMCase polyAb (Product code: ab72309, abcam) at a dilution of 1:200–1:400. In addition, the HRP-conjugated secondary antibodies (Santa cruz biotechnologies) and DAB Substrate kit (Cat: DA1010, Solabrio, life science) were used for color development.

For western blotting, the footpad tissues of nu/nu-BALB/c mice and spleen tissues of BALB/c mice infected with *F*. *pedrosoi*-hyphae were harvested respectively at 10 days, 30 days, 50 days and 80 days post-inoculation. Protein extracts were prepared from tissues by homogenizing on ice using RIPA buffer (Sigma-aldrich, R0278) added with 1×protease inhibitor cocktails (Thermo, 78410) and 1 mM phenylmethylsuphonylfluoride (PMSF) (Sigma-aldrich, P7626). Then the total protein was quantified via Bicincho Acid Assay (Pierce), denatured with 2× laemmli buffer (Sigma-aldrich, 38733) and heated to 95°C for 5 min. 20 μg of total protein was loaded onto each well of a 7.5% polyacrylamide gel (BioRad), separated via electrophoresis, and transferred to PVDF membrane (Sigma-aldrich, P2438). The membrane was blocked with 5% milk-TBS-Tween buffer and probed overnight with rabbit-derived Anti-AMCase polyAb (Product code: ab72309, abcam) at a dilution of 1:1000, and then with HRP-conjugated anti-rabbit IgG (Cat: #7071, Cell Signaling Technology) at a dilution of 1:2000. Finally, the blots were developed using the chemiluminescence reagents ECL Plus, and shown on Hyper Film (Amersham, GE Healthcare).

### Detection of Th1/Th2/Th17-related cytokines via mouse CBA kit

For infection with live *F*. *pedrosoi*, the BALB/c mice were randomly divided into three experimental groups and an inoculation control group (n = 12 per group). The mice in the experimental groups were respectively infected intraperitoneally with 500 μl of *F*. *pedrosoi*-spores, -hyphae or in vitro-induced sclerotic cells at a concentration of 5.0×10^8^/mL. In the inoculation control group, the mice of the same age were simultaneously inoculated intraperitoneally with 500 μl of normal saline. Single-cell suspensions of splenocytes were prepared respectively from the BALB/c mice in the three experimental groups as well as the inoculation group at 10 days, 30 days and 50 days post inoculation (n = 4 per group at each indicated time point). Before measurement, the cells were adjusted to 2×10^6^ cells in a volume of 2 mL RPMI1640 plus 10% FCS and were treated with 6 μl of Cell Stimulation Cocktail (500×) (Cat: 00–4970, eBioscience) at 37°C for 6h.

For stimulation with heat-killed *F*. *pedrosoi*-sclerotic cells induced in vitro, the BALB/c mice were randomly divided into two experimental groups and one inoculation control group (n = 16 per group). The mice in the experimental groups were respectively inoculated intraperitoneally with 500 μl of heat-killed sclerotic cells (5.0×10^8^/mL) for 3 times at 7-day intervals in the presence or absence of chitinase treatment. In the inoculation control group, the mice were simultaneously inoculated intraperitoneally with 500 μl of normal saline. Single-cell suspensions of splenocytes were prepared respectively from the BALB/c mice in the experimental and control groups at 7 days, 14 days, 21 days and 36 days after initial inoculation (n = 4 per group at each indicated time point). Before measurement, the cells were adjusted to 2×10^6^ cells in a volume of 2 mL RPMI1640 plus 10% FCS and were treated with 6 μl of Cell Stimulation Cocktail (500×) (Cat: 00–4970, eBioscience) at 37°C for 6h.

Afterwards, BD Cytometric Beads Array (CBA) murine cytokine kit was introduced to measure the Th1/Th2/Th17 cytokine levels in culture supernatants of splenocytes prepared as above-mentioned. Briefly, seven bead populations with distinct APC-A fluorescence intensities had been coated with capture Abs specific for IL-2, IL-4, IL-6, IFN-γ, TNF-α, IL-17A and IL-10 proteins, as indicated by A1-A7 in turns. The concentration of specific cytokine mentioned above can be revealed by the fluorescence intensity of PE-conjugated detection Ab, and be calculated according to the standard curve established by cytokine standards using FCAP Array software. The assay diluent incubated with beads and PE-conjugated Ab was set as blank control.

### Statistical analysis

Data are presented as mean ± standard error of the mean (SEM). Statistical comparisons between groups were performed using Univariate Analysis of Variance (ANOVA) and the LSD-t test. A p-value<0.05 was considered as significant, and p-value<0.01 as highly significant. Statistical graphs were drawn by GraphPad Prism Software in the present study.

## Results

### Viability of fungal inocula

**Fig 1:**
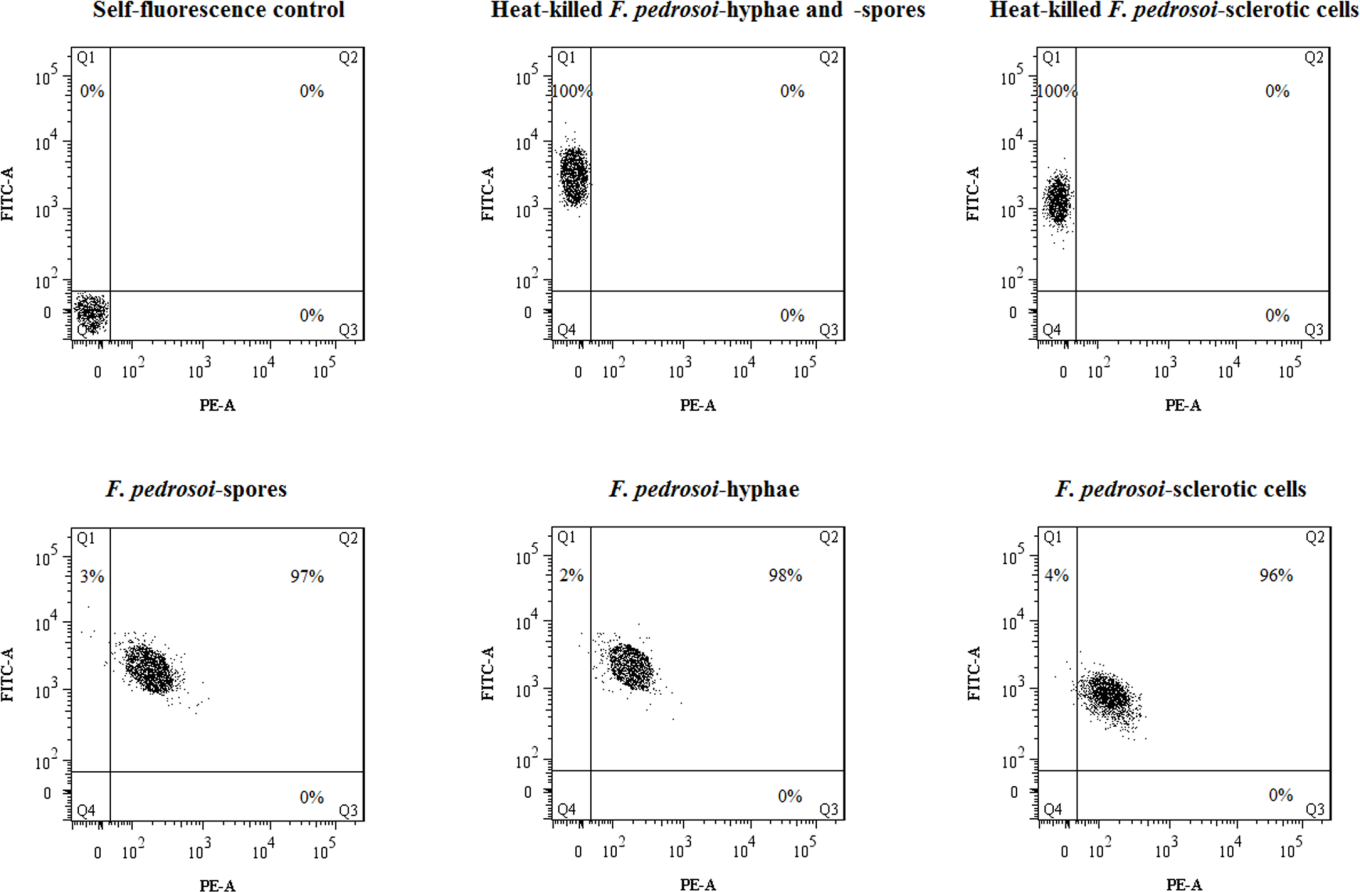
The viability test of *F*. *pedrosoi*-spores, -hyphae and induced sclerotic cells. The *F*. *pedrosoi*-spores, -hyphae and induced sclerotic cells were pre-stained with FUN1 Cell Stain (Cat: F7030, Invitrogen), a unique two-color fluorescent viability probe for yeast and fungi, and the viability was measured using flow cytometry assay according to the protocol by setting the 488 nm laser line as the excitation source, standard FITC channel (FITC-A) for detection of green emission (dead/metabolically inactive cells) and PE channel (PE-A) for red emission (live/metabolically active cells). The fungal cells with red emission or two-color emissions were considered viable (Percentage in Q2 and Q3 regions). In addition, the fungal cells without FUN1 treatment were set as self-fluorescence control. The fungal cells which were heat-killed by incubation in a water-bath at 70°C for 90 min and stained with FUN1 were set as the dead cell control.

For saprophytic *F*. *pedrosoi*-spores, -hyphae or in vitro-induced sclerotic cells prepared in this study, the viability rate of each fungal cell type reached 96% or above (Fig 1, Q2 and Q3 regions).

### Self-healing infection was observed in BALB/c mice which were intraperitoneally inoculated with *F*. *pedrosoi*-spores

**Fig 2:**
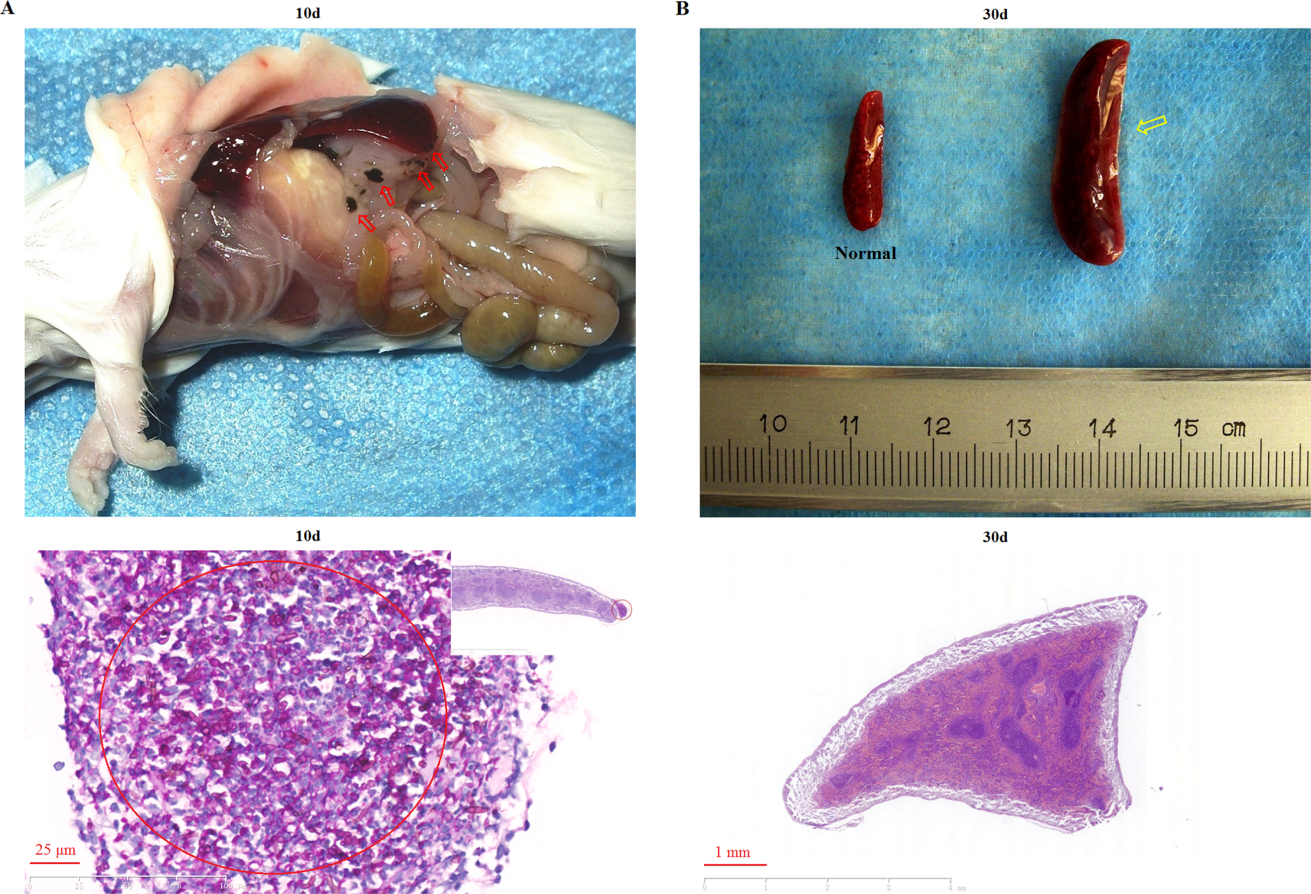
Intraperitoneal infection of BALB/c mice with *F*. *pedrosoi*-spores developed to be self-healing. (A) Infectious foci in the abdomen at 10 days post-inoculation, as indicated by red arrows (top panel); PAS staining (×400) for the infected spleen was performed, and the majority of spores with budding were indicated by red circle (bottom panel). (B) Spleens from BALB/c mice at 30 days post-inoculation and normal control, scale bar = 1 mm (top panel); PAS staining (×400) for the spleen from BALB/c mice at 30 days post-inoculation (bottom panel).

For mice intraperitoneally inoculated with *F*. *pedrosoi*-spores, scattered small black or dark-brown nodules on the abdomen formed and distributed to the bowel, stomach and spleen in the first 10 days post-inoculation (Fig 2A, top panel). Clinical improvement was manifest by gradual absorption of infectious foci with absolute elimination of this agent, and a significant increase in spleen volume was observed in contrast with normal controls till day 30 post-inoculation (Fig 2B, top panel). Histological sections showed well-encapsulated abscesses in the infected spleen where the inoculated agents still remained as spore morphology and were surrounded by numerous neutrophils at day 10 post-inoculation (Fig 2A, bottom panel). Infectious foci were not observed in the spleen at day 30 post-inoculation (Fig 2B, bottom panel). The original spore inoculums were not recovered from fungal culture of the spleens or other abdominal tissues isolated from mice at 30 days post-inoculation or later.

### Recalcitrant infection was observed in BALB/c mice intraperitoneally inoculated with *F*. *pedrosoi*-hyphae with in vivo transformation of this agent into sclerotic cells

**Fig 3:**
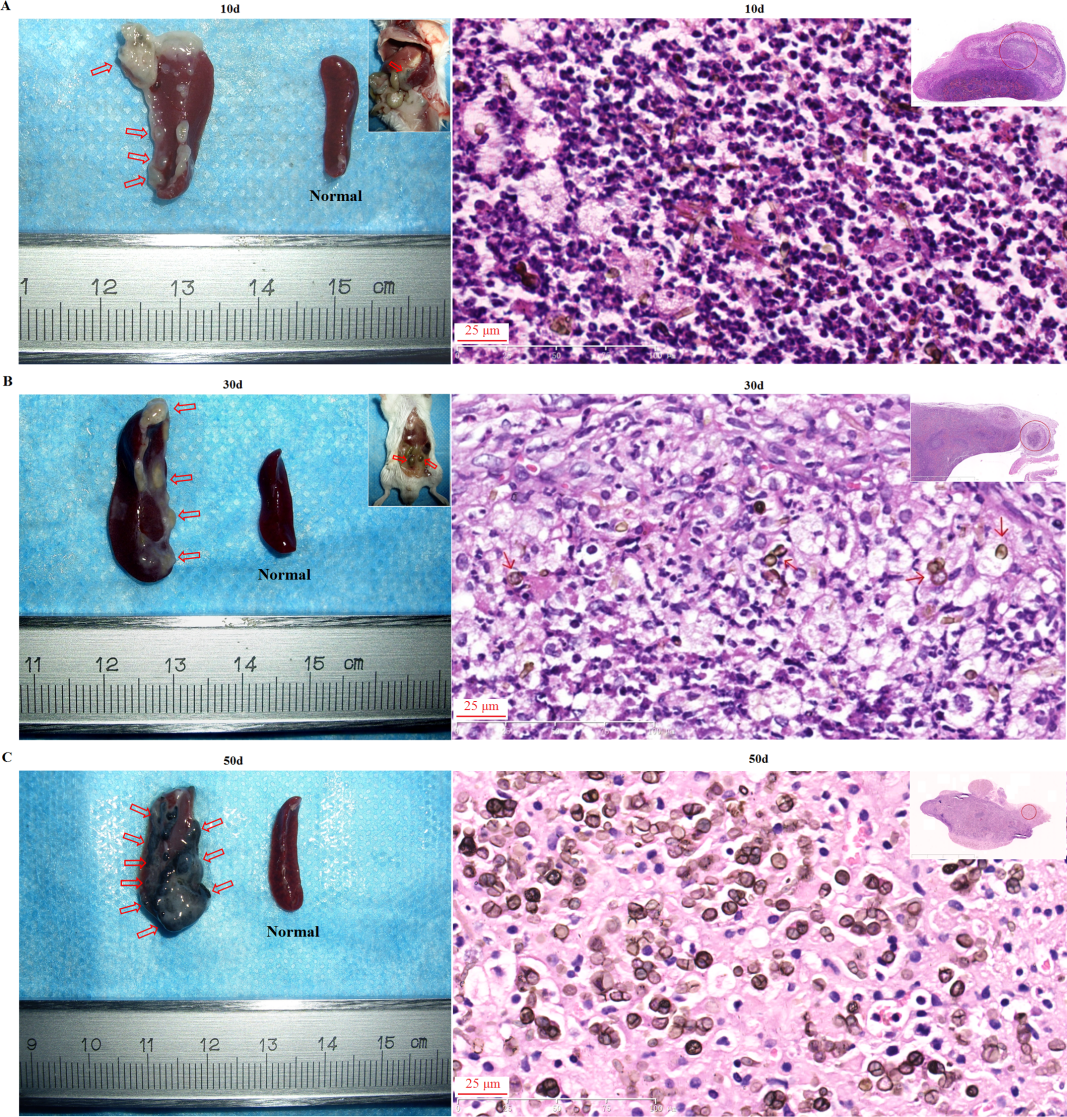
Recalcitrant infection of BALB/c mice was induced by intraperitoneal injection of *F*. *pedrosoi* hyphae with the formation of sclerotic cells in the nidus. (A-C) Spleens respectively from BALB/c mice at 10 days (A), 30 days (B) and 50 days (C) post-inoculation as well as the normal control (left column). The infectious foci in the spleens and abdomen were indicated by red arrows. Scale bar = 1 mm. (A-C) HE staining (×400) for the infected spleens at 10 days (A), 30 days (B) and 50 days (C) post-inoculation (right column).

For mice intraperitoneally inoculated with *F*. *pedrosoi*-hyphae, the affected spleen and stomach were attached together by inflammatory peritoneum, and multiple purulent nodules began to form in the spleen in the first 10 days post-inoculation and then disseminated to the peritoneal serosa until 30 days post-inoculation (Fig 3A and 3B, left panels). Multiple yellow-white purulent nodules gradually transformed into black nodular lesions which further disseminated throughout the spleen during the next part of the 50-day observation period following inoculation (Fig 3C, left panel). In addition, significant increase in spleen volume of intraperitoneally infected BALB/c mice was consecutively observed in contrast with normal controls at 10, 30 and 50 days post-inoculation (Fig 3A–3C, left panels). Histological sections showed predominant neutrophil infiltration into the spleen where the inoculated *F*. *pedrosoi* remained as hyphal form at 10 days post-inoculation (Fig 3A, right panel). However, with transformation of the inoculated *F*. *pedrosoi*-hyphae into sclerotic cells, a significant decrease or absence of neutrophil recruitment was consecutively observed at 30 days and 50 days post-inoculation (Fig 3B and 3C, right panels). The original hyphae inoculums were recovered from fungal culture of the spleens or other abdominal tissues isolated from BALB/c mice at 50 days post-inoculation and have 100% sequence identity at “ITS1-5.8S-ITS2” region with *F*. *pedrosoi* WH10-002 (GenBank no: GQ420654.1).

### Transformation of saprophytic *F*. *pedrosoi* into sclerotic cells was induced in vitro

**Fig 4:**
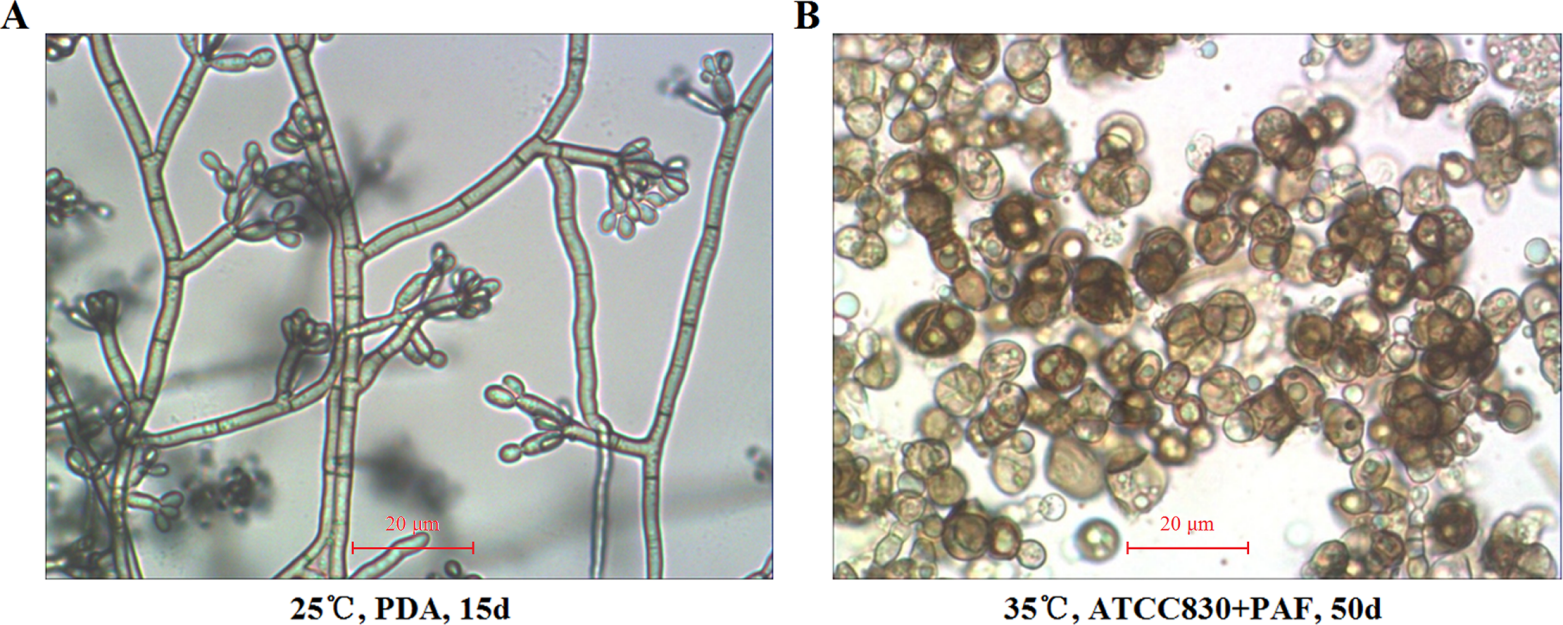
In vitro induced transformation of saprophytic *F*. *pedrosoi* into sclerotic cells. Optical microscope was used to characterize the morphology of saprophytic *F*. *pedrosoi* growing on PDA (A) and in-vitro transformed sclerotic cells cultured in ATCC830 medium plus PAF for 50 days (×400) (B). Scale bar = 20 μm (A and B).

The slide culture of *F*. *pedrosoi* WH10-002 growing on PDA at 25°C for 15 days showed characteristic dematiaceous hyphae originating terminal cylindrical conidiophores with small subhyaline conidia produced by acropetal budding, and sympodially arranged on short denticles (Fig 4A). Morphological transformation of this agent from its hyphal form into brownish, multi-septated sclerotic cells was observed in ATCC 830 medium plus 10^−6^ M PAF at 35°C within 50 days post-inoculation (Fig 4B).

### Recalcitrant infection was also observed in BALB/c mice which were intraperitoneally inoculated with in vitro-induced *F*. *pedrosoi*-sclerotic cells

**Fig 5:**
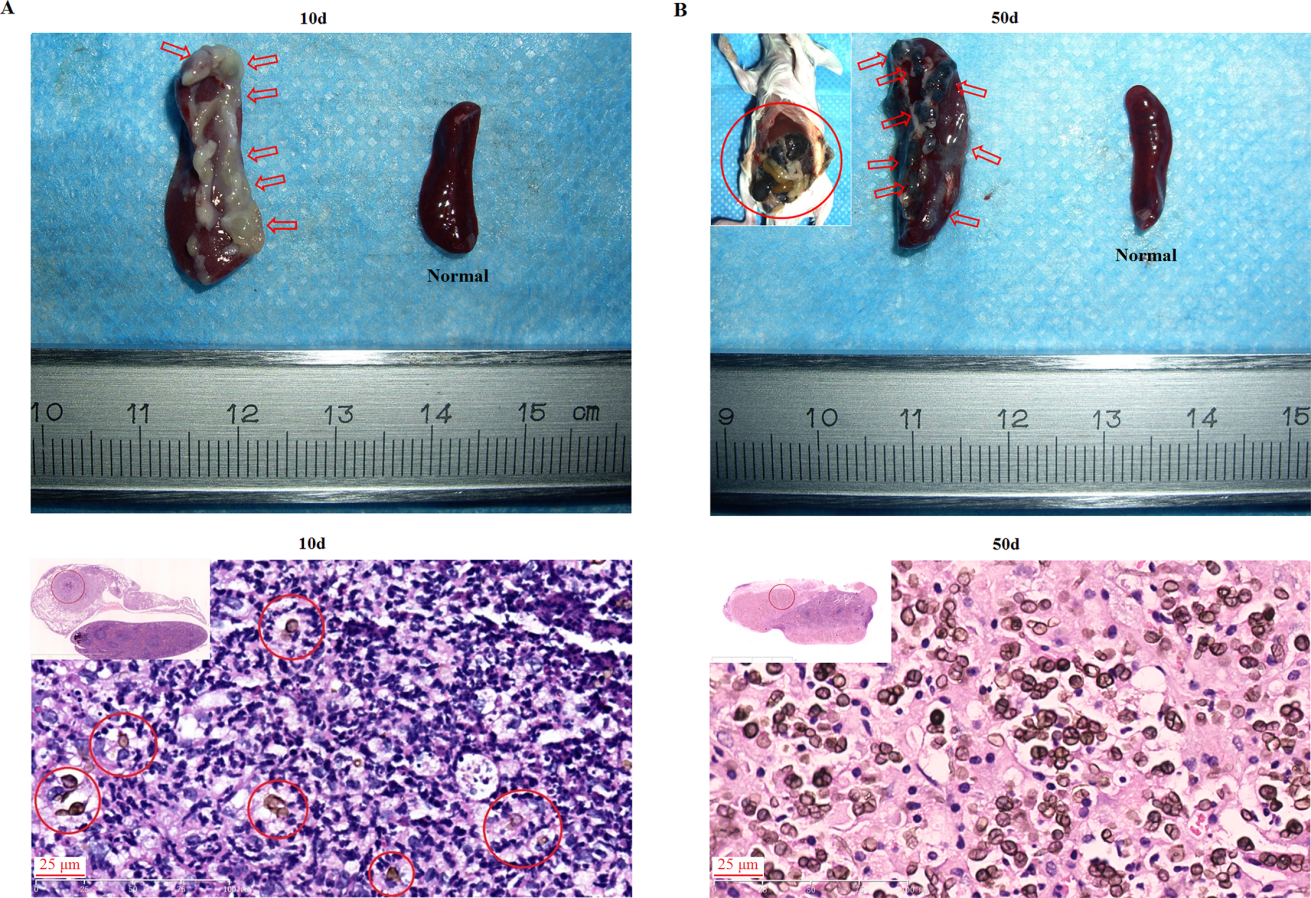
Recalcitrant infection of BALB/c mice was induced by intraperitoneal injection of in vitro-transformed *F*. *pedrosoi* sclerotic cells. (A and B) Spleens respectively from BALB/c mice at 10 days (A) and 50 days (B) post-inoculation as well as the normal control (top row). The infectious foci in the spleens and abdomen were indicated by red arrows and red circle. Scale bar = 1 mm. (A and B) HE staining (×400) for infected spleens at 10 days (A) and 50 days (B) post-inoculation (bottom row).

For mice intraperitoneally inoculated with in vitro induced *F*. *pedrosoi*-sclerotic cells, multiple yellow-white, purulent nodules formed and adhered with each other in the spleen in the first 10 days post-inoculation, gradually transforming into black nodular lesions which coalesced and disseminated to the peritoneal serosa and the stomach, bowel, liver and other abdominal organs within the 50-day observation period following inoculation (Fig 5A and 5B, top panels). In addition, significant increase in spleen volume of the intraperitoneally infected BALB/c mice was consecutively observed at 10 days and 50 days post-inoculation (Fig 5A and 5B, top panels). Histological sections showed formation of micro-abscess in which the inoculated sclerotic cells were surrounded by neutrophils at 10 days post-inoculation (Fig 5A, bottom panel). However, the disappearance of infiltrated neutrophils was observed in the infected spleen with the dissemination of sclerotic cells at 50 days post-inoculation (Fig 5B, bottom panel). The sclerotic cell inoculums were recovered from fungal culture of the infected spleens or other abdominal tissues at 50 days post-inoculation and have 100% sequence identity at “ITS1-5.8S-ITS2” region with *F*. *pedrosoi* WH10-002 (GenBank no: GQ420654.1).

### An early and persistent increase in IFN-γ production was observed in the splenocytes from BALB/c mice intraperitoneally inoculated with *F*. *pedrosoi*-spores

**Fig 6:**
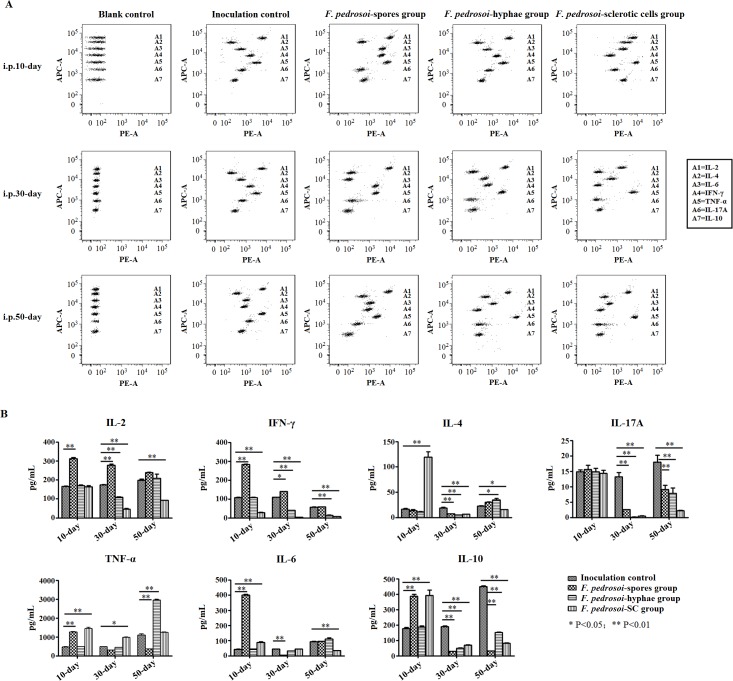
IFN-γ production was initially inhibited, followed by pan-inhibition of Th1/Th2/Th17 cytokines production in splenocytes of BALB/c mice intraperitoneally infected with *F*. *pedrosoi* sclerotic cells. (A and B) BD Cytometric Beads Array (CBA) murine cytokine kit was introduced to measure the Th1/Th2/Th17 cytokine levels in culture supernatants of splenocytes from BALB/c mice at 10 days, 30 days and 50 days after intraperitoneally inoculation with *F*. *pedrosoi*-spores, -hyphae or transformed sclerotic cells respectively (n = 12 per group). The BALB/c mice of the same age intraperitoneally inoculated with normal saline were set as inoculation control (n = 12). Before measurement, the splenocytes from BALB/c mice were adjusted to 2×10^6^ cells in a volume of 2 mL RPMI1640 plus 10% FCS, and were pretreated with 1×Cell Stimulation Solution at 37°C for 6h. (A) Seven bead populations with distinct APC-A fluorescence intensities had been coated with capture Abs specific for IL-2, IL-4, IL-6, IFN-γ, TNF-α, IL-17A and IL-10 proteins, as indicated by A1-A7 in turns. The concentration of specific cytokine mentioned above can be revealed by the fluorescence intensity of PE-conjugated detection Ab, and be calculated according to the standard curve established by cytokine standards using FCAP Array software. The assay diluent incubated with beads and PE-conjugated Ab was set as blank control. (B) Graph showing the concentration (pg/mL) of Th1/Th2/Th17 cytokines produced by splenocytes from uninfected and *F*. *pedrosoi*-infected BALB/c mice mentioned above at the indicated days post-inoculation. Data represent the mean±SEM (n = 4 per group at each indicated time point) and statistical analysis was performed using Univariate ANOVA and LSD-t test. Significant: ^*^ P<0.05; Highly Significant: ^**^ P<0.01.

For mice intraperitoneally inoculated with *F*. *pedrosoi*-spores, significantly increased IL-2, INF-γ, TNF-α, IL-6 and IL-10 levels were observed in the culture supernatant of splenocytes at 10 days post-inoculation in comparison with the inoculation control group (LSD-t test, p<0.01) (Fig 6A and 6B). Furthermore, increased levels of IL-2 and IFN-γ, and decreased levels of IL-4, IL-17A, IL-6 and IL-10 were simultaneously detected in this group at 30 days post-inoculation in comparison with the inoculation control group (LSD-t test, p<0.05 or p<0.01) (Fig 6A and 6B). At 50 days post-inoculation, significantly decreased levels of IL-17A, TNF-α and IL-10 were observed in this group in comparison with the inoculation control group (LSD-t test, p<0.01) (Fig 6A and 6B). During the 50-day observation period following inoculation, the agents in tissue remained as spore morphology until elimination without transformation into hyphae or sclerotic cells.

### Decreased production of IFN-γ and IL-17A was detected in the splenocytes from BALB/c mice with transformation of *F*. *pedrosoi*-hyphae into sclerotic cells in spleen tissue

For mice intraperitoneally inoculated with *F*. *pedrosoi*-hyphae, no significant differences were observed in IL-2, INF-γ, IL-4, IL-17A, TNF-α, IL-6 and IL-10 levels in the culture supernatant of splenocytes in comparison with the inoculation control group at 10 days post-inoculation when the inoculated agents remained as hyphal form in tissue (Figs 3A, 6A and 6B). However, significantly decreased levels of IL-2, IFN-γ, IL-4, IL-17A and IL-10 were detected when compared with the inoculation control group at 30 days post-inoculation with transformation of this agent into sclerotic cells in spleen tissue (LSD-t test, p<0.01) (Figs 3B, 6A and 6B). At 50 days post-inoculation, when the sclerotic cells disseminated throughout the spleen, significantly decreased levels of IFN-γ and IL-17A as well as IL-10, and increased levels of IL-4 and TNF-α were detected in comparison with the inoculation control group (LSD-t test, p<0.05 or p<0.01) (Figs 3C, 6A and 6B).

### An early decrease in IFN-γ production was observed in the splenocytes from BALB/c mice intraperitoneally inoculated with in vitro-induced *F*. *pedrosoi*-sclerotic cells, followed by pan-inhibition of Th1/Th2/Th17 cytokines production

For this group of mice, an early decreased IFN-γ level and simultaneously increased levels of IL-4, IL-6, TNF-α and IL-10 were detected in the culture supernatant of splenocytes at 10 days post-inoculation in comparison with the inoculation group (LSD-t test, p<0.01) (Fig 6A and 6B). However, other than an increased level of TNF-α at 30 days post-inoculation, significantly decreased levels of Th1/Th2/Th17 cytokines including IL-2, IFN-γ, IL-4, IL-17A, as well as IL-10 were detected at both 30 days and 50 days post-inoculation (LSD-t test, p<0.05 or p<0.01) (Fig 6A and 6B). During the 50-day observation period following inoculation, the agents remained as sclerotic cell form in the spleen tissue (Fig 5A and 5B, bottom panels).

### Intraperitoneal administration of rmIFN-γ effectively reduces the fungal load in the infected mouse spleen and dampens peritoneal dissemination of *F*. *pedrosoi*-sclerotic cells

**Fig 7:**
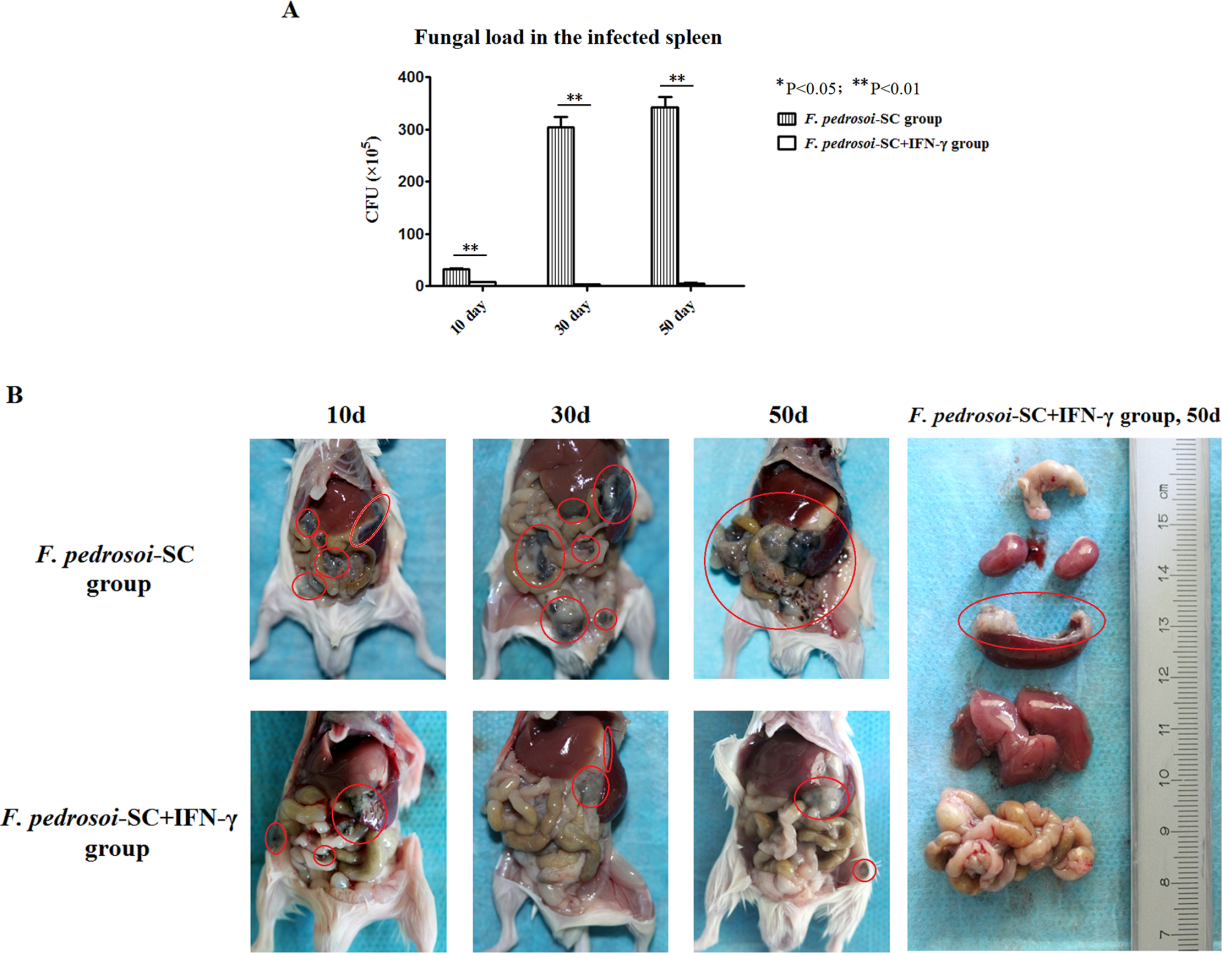
Intraperitoneal administration of IFN-γ effectively reduced fungal load in the infected mice spleens, and dampened peritoneal dissemination of *F*. *pedrosoi*-sclerotic cells. Abbreviations: *F*. *pedrosoi*-SC (*F*. *pedrosoi*-sclerotic cells); *F*. *pedrosoi*-SC+IFN-γ (*F*. *pedrosoi*-sclerotic cells and exogeneous IFN-γ) (A) The fungal load in the infected spleens of BALB/c mice respectively at 10 days, 30 days and 50 days post-inoculation was compared between the group intraperitoneally injected with *F*. *pedrosoi*-SC and that injected with *F*. *pedrosoi*-SC+IFN-γ. Data represent the mean±SEM (n = 4 per group at each indicated time point) and statistical analysis was performed using Univariate ANOVA and LSD-t test. The Significant: ^*^ P<0.05; Highly Significant: ^**^ P<0.01. (B) The peritoneal dissemination of *F*. *pedrosoi*-SC in BALB/c mice was compared between the group intraperitoneally injected with *F*. *pedrosoi*-SC and that with *F*. *pedrosoi*-SC+IFN-**γ** respectively at 10 days, 30 days and 50 days post-inoculation. The abdominal and retroperitoneal organs including the colon, kidneys, spleen, liver and bowels were respectively isolated from the mouse injected with *F*. *pedrosoi*-SC+IFN-**γ** at 50 days post-inoculation for fungal examination (from top to bottom, right panel). The infectious foci were indicated by red circles.

For the group of mice intraperitoneally injected with *F*. *pedrosoi*-sclerotic cells and exogeneous rmIFN-γ, the fungal load in the whole spleen was significantly lower than that in the group intraperitoneally injected merely with *F*. *pedrosoi*-sclerotic cells respectively at 10 days, 30 days and 50 days post-inoculation (Fig 7A).

Furthermore, abdominal anatomy showed that in the group injected merely with *F*. *pedrosoi*-sclerotic cells, multiple small black or dark-brown nodules formed and scattered in the bowels, liver and coalesced into strip-like lesions along the border of spleen at 10 days post-inoculation (Fig 7B). Subsequently, the black nodular lesions enlarged significantly in the infected spleen at 30 days post-inoculation, and further disseminated into the whole abdominal viscera including the spleen, stomach, liver, bowels and peritoneal serosa at 50 days post-inoculation (Fig 7B). In contrast, for the group of mice injected with *F*. *pedrosoi*-sclerotic cells and exogeneous rmIFN-γ, although the scattered distribution of dark-brown nodules or plaque were seen in the bowels and spleen at 10 days post-inoculation, only a few gray-white, purulent nodules were localized on the spleen and the original inoculation site at 30 days and 50 days post-inoculation (Fig 7B).

### When compared with *F*. *pedrosoi*-spores and -hyphae, intraperitoneal infection with in vitro-transformed sclerotic cells induced relatively lower neutrophil ROS generation in the infected spleens of BALB/c mice at 10 days post-inoculation

**Fig 8:**
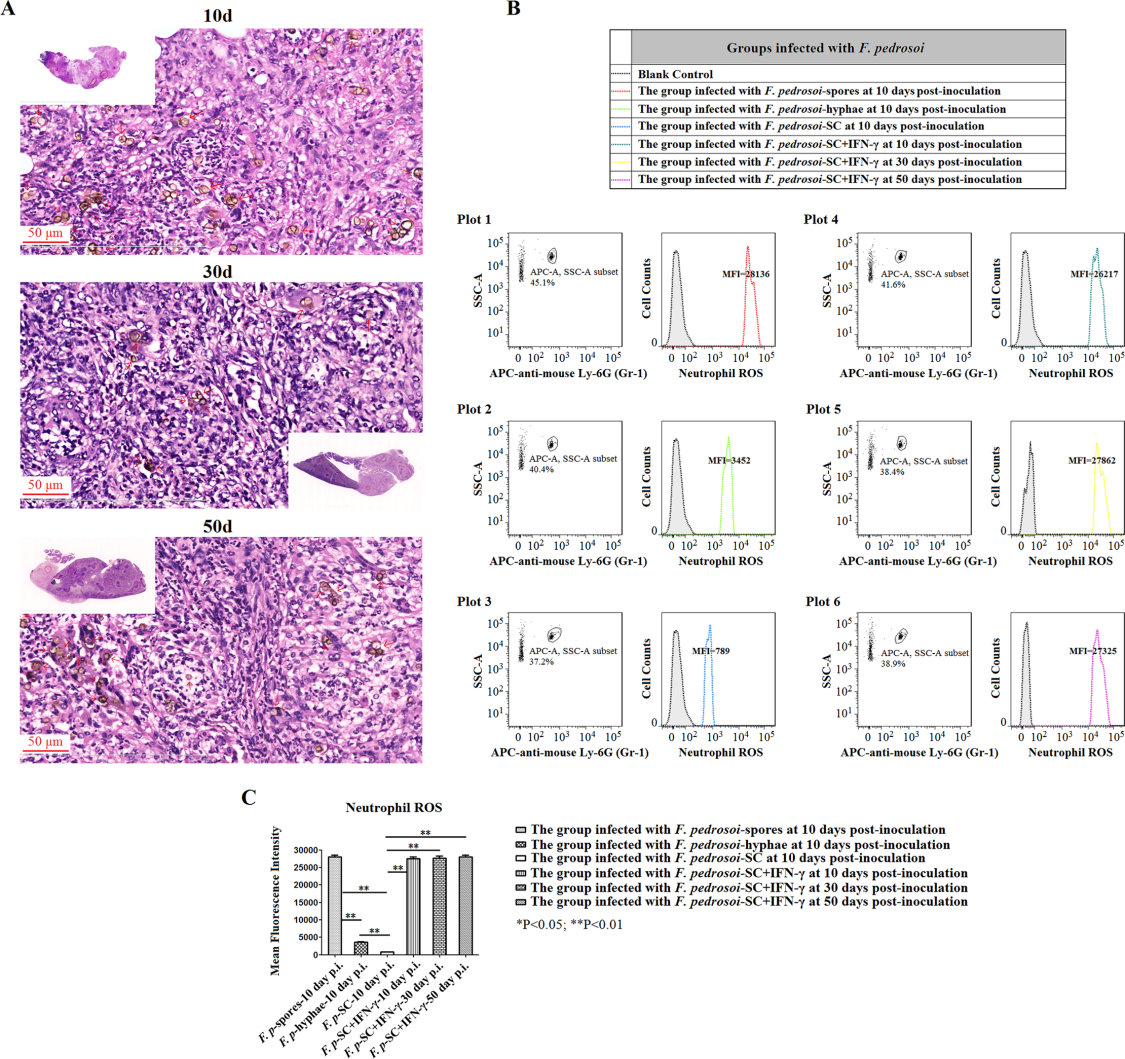
Exogeneous IFN-γ contributed to the formation and maintenance of micro-abscess, and restored the decrease in neutrophil ROS production in the mouse spleen infected with *F*. *pedrosoi*-sclerotic cells. Abbreviations: *F*. *pedrosoi*-SC (*F*. *pedrosoi*-sclerotic cells); *F*. *pedrosoi*-SC+IFN-γ (*F*. *pedrosoi*-sclerotic cells and exogeneous IFN-γ) (A) HE staining (×400) for the infected spleens from the BALB/c mice intraperitoneally inoculated with *F*. *pedrosoi*-SC or with *F*. *pedrosoi*-SC+IFN-γ respectively at 10 days, 30 days and 50 days post-inoculation. The sclerotic cells with transverse septation in the infectious foci were indicated by red arrows. (B and C) Neutrophil ROS was measured in the infected spleens from the BALB/c mice intraperitoneally injected with *F*. *pedrosoi*-spores, -hyphae, -SC, and -SC+IFN-γ at the indicated days post-inoculation. (B) Flow cytometry assay was introduced to measure ROS generation in neutrophils. Briefly, the infected nodules in the spleens were aseptically isolated, and the neutrophils were selected by using side scatter (SSA) and APC-conjugated anti-mouse Ly-6G (Gr-1) mAb. The cells incubated with APC-conjugated rat-derived IgG2a kappa were set as isotype control. ROS levels in neutrophils were measured using 2’, 7’-dichlorodihydrofluorescin diacetate (DCHF-DA) as fluorescence probe, and were represented as mean fluorescence intensity (MFI). The neutrophils without adding DCHF-DA were set as blank control. (C) Neutrophil ROS in the infected spleens was compared among the groups of BALB/c mice mentioned above at indicated days post-inoculation. Data represent the mean±SEM (n = 4 per group at each indicated time point) and statistical analysis was performed using Univariate ANOVA and LSD-t test. Highly Significant: ^**^ P<0.01.

For mice infected with in vitro-transformed *F*. *pedrosoi*-sclerotic cells, neutrophil influx was observed in the infected spleen at 10 days post-inoculation (Fig 5A), but the level of neutrophil ROS production was significantly lower than those of the *F*. *pedrosoi*-spores and -hyphae groups in which the inoculated agents still remained as saprophytic morphology at that time (LSD-t test, p<0.01) (Figs 2A, 3A, 8B and 8C).

### Exogeneous rmIFN-γ contributed to the formation and maintenance of micro-abscess and restored the decrease in neutrophil ROS generation in the mouse spleen infected with *F*. *pedrosoi*-sclerotic cells

For mice intraperitoneally injected with *F*. *pedrosoi*-sclerotic cells and rmIFN-γ, histological sections from the infected spleens at 10 days, 30 days and 50 days post-inoculation consistently showed the formation and maintenance of micro-abscess in which the sclerotic cells were surrounded by infiltrating neutrophils and histocyte-like cells (Fig 8A). In contrast, for mice intraperitoneally injected merely with *F*. *pedrosoi*-sclerotic cells, although the histological sections showed the formation of micro-abscess at 10 days post-inoculation (Fig 5A), the disappearance of infiltrating neutrophils was observed in the infected spleen with the dissemination of sclerotic cells at 50 days post-inoculation (Fig 5B).

Furthermore, upon intraperitoneal administration of murine IFN-γ, the decreased neutrophil ROS production in the spleen of mice infected with *F*. *pedrosoi*-sclerotic cells was completely restored to the ROS level of the group intraperitoneally infected with *F*. *pedrosoi*-spores at 10 days post-inoculation, and maintained at this level at 30 days and 50 days post-inoculation (Fig 8B and 8C).

### It is the chitin-like component, but not the β-glucans or mannose moiety, that exclusively accumulates on the surface of in vitro-transformed *F*. *pedrosoi*-sclerotic cells

#### Confocal microscope examinations

**Fig 9:**
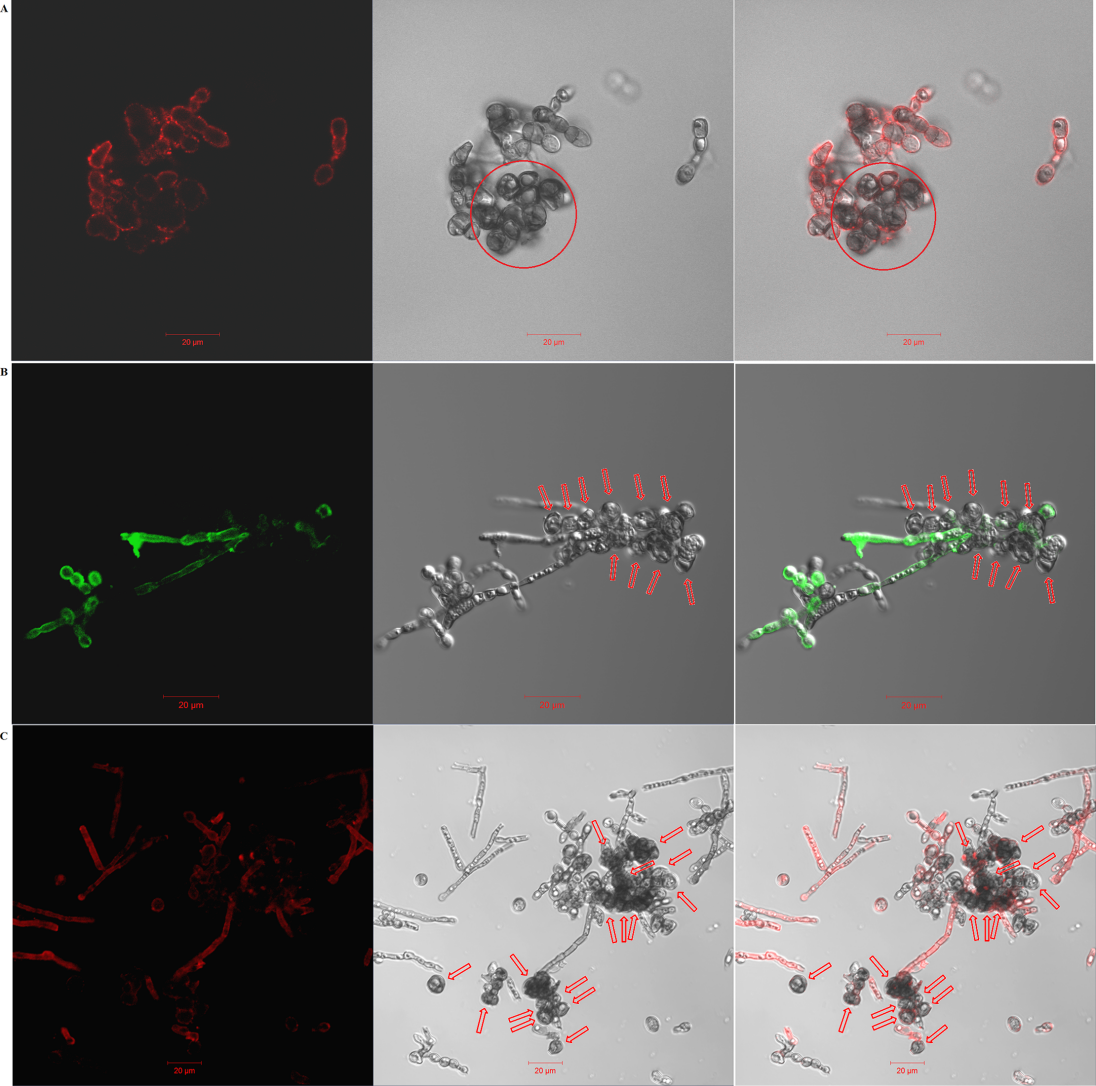
Dectin-1, MR and Dectin-2 mainly bind to the surface of *F*. *pedrosoi-*hyphae, but not the transformed sclerotic cells. (A-C) The binding of murine-derived Dectin-1, Mannose Receptor (MR), and Dectin-2 to *F*. *pedrosoi* cultured in ATCC 830 medium with 10^−6^ M PAF at 35°C for 30 days was detected respectively by PE-conjugated anti-murine Dectin-1 mAb (A), FITC-conjugated anti-murine MR mAb (B), and PE-conjugated anti-murine Dectin-2 mAb (C) using confocal microscope. Left column: fluorescence field; Middle column: bright field; Right column: merged images. Scale bar = 20 μm. The transformed sclerotic cells with cross-septation and swelling chlamydospores were indicated by red circle (A) or red arrows (B and C). The fungal cells incubated only with PE-conjugated anti-murine Dectin-1 or Dectin-2 mAb, or only with FITC-conjugated anti-murine MR mAb were set as blank control.

**Fig 10:**
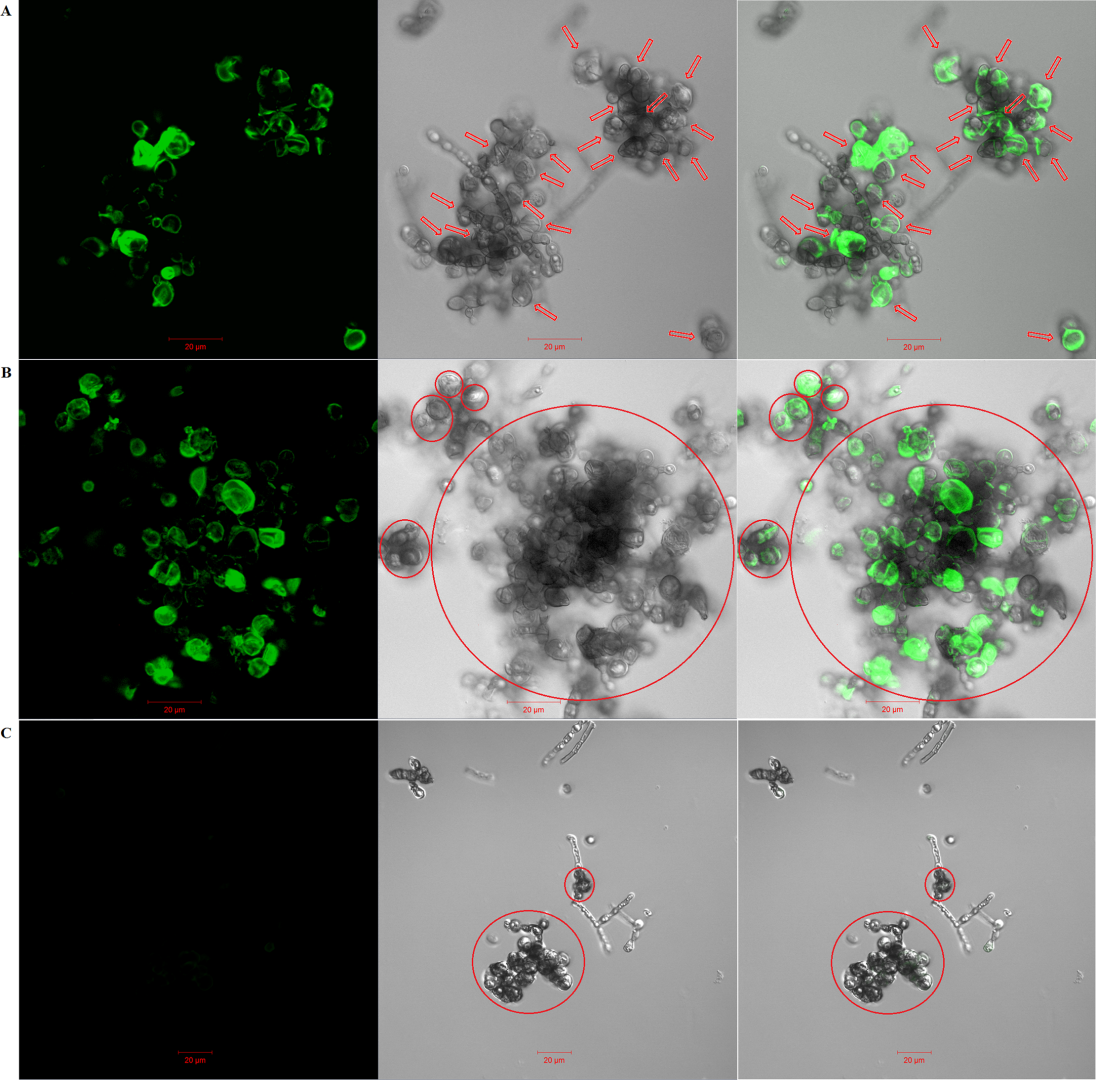
Some chitin-like component exclusively accumulates on the surface of in-vitro transformed sclerotic cells and hyphal tip. (A-C) FITC-conjugated Wheat Germ Agglutinin (WGA), a lectin that specifically binds to chitin, was introduced to detect chitin distribution on the surface of *F*. *pedrosoi* using confocal microscope. Left column: fluorescence field; Middle column: bright field; Right column: merged images. The binding of FITC-conjugated WGA to *F*. *pedrosoi* cultured in ATCC 830 medium with 10^−6^ M PAF for 40 days (A) or 50 days (B) was detected using confocal microscope. (C) *F*. *pedrosoi* cultured in ATCC 830 medium with 10^−6^ M PAF for 40 days with chitinase pretreatment was set as control. Scale bar = 20 μm. The majority of transformed sclerotic cells with cross-septation as well as swelling chlamydospores were indicated by red arrows (A) and circles (B and C).

**Fig 11:**
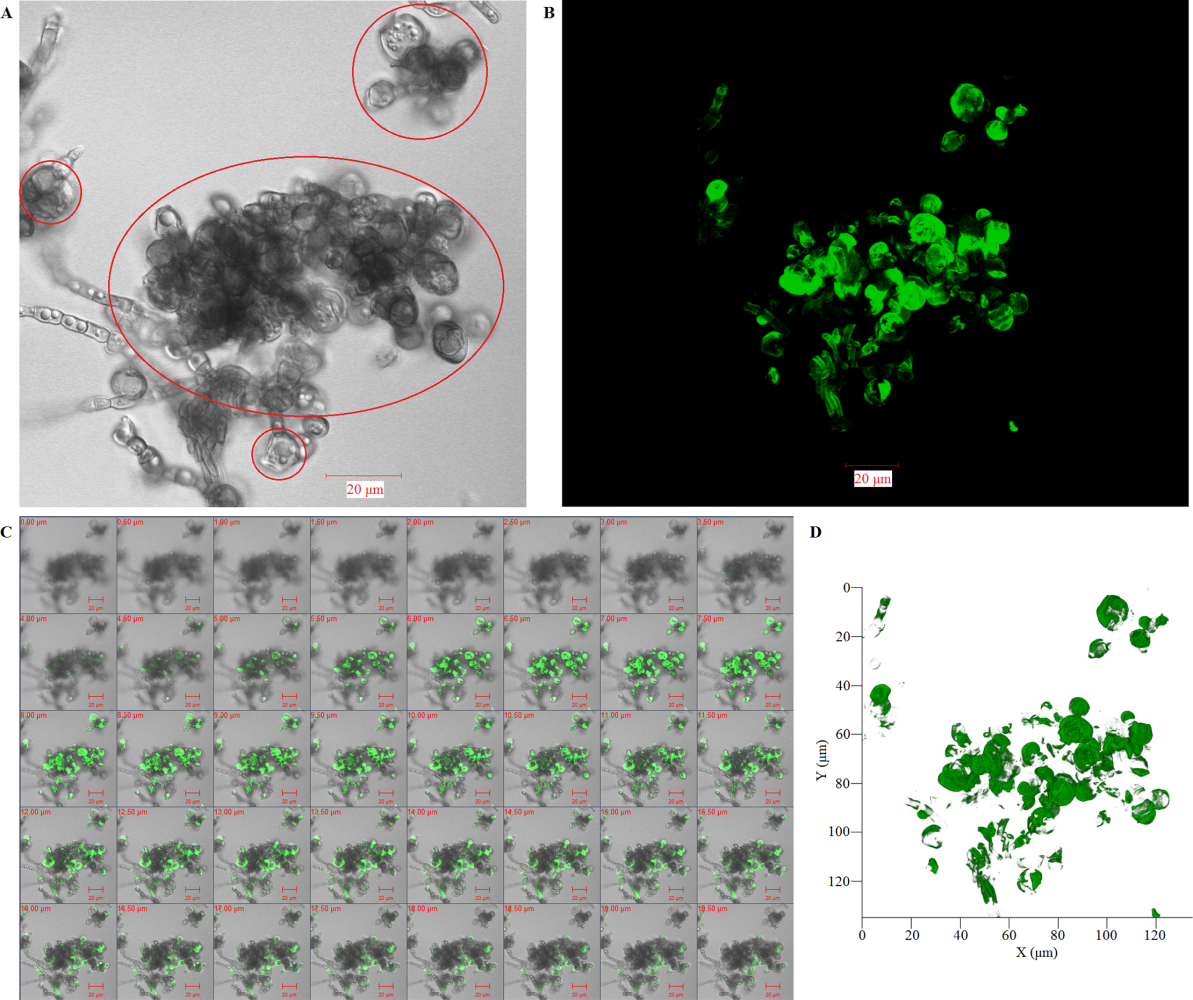
Three-dimensional conformation of chitin distribution re-constructs the figuration of in-vitro transformed sclerotic cells. (A-D) Three-dimensional distribution of chitin on the surface of *F*. *pedrosoi* cultured in ATCC 830 medium with 10^−6^ M PAF for 40 days were analyzed by FITC-conjugated WGA using confocal tomoscanning. (A) *F*. *pedrosoi* in the bright field. In-vitro transformed sclerotic cells with cross-septation were indicated by red circles. (B) Three-dimensional conformation of chitin distribution on *F*. *pedrosoi* was represented by FITC-conjugated WGA in the fluorescence field. (C) The binding of FITC-conjugated WGA on the surface of *F*. *pedrosoi* was analyzed by confocal tomoscanning. Each cross-section thickness was set as 0.5μm. Scale bar = 20 μm. (D) Three-dimensional conformation of chitin distribution represented by FITC-conjugated WGA was reconstructed according to confocal tomoscanning.

For the *F*. *pedrosoi* cultured in ATCC 830 medium with 10^−6^ M platelet activating factor (PAF) at 35°C for 30 days, we observed that the binding site of murine-derived Dectin-1 was largely distributed over the surface of hyphae, except for a little speckle-like distribution on the transformed sclerotic cells with cross-septation (Fig 9A). Similarly, the binding sites of both murine-derived mannose receptor (MR) and Dectin-2 were exclusively distributed over the surface of hyphae, but not over the chlamydospores or transformed sclerotic cells with cross-septation (Fig 9B and 9C). However, for the *F*. *pedrosoi* cultured in ATCC 830 medium with 10^−6^ M PAF at 35°C for 40 days or 50 days, we observed that the binding site of WGA was mainly distributed over the surface of a majority of transformed sclerotic cells with cross-septation, as well as the terminal part of growing hyphae (Fig 10A and 10B). In addition, the binding of WGA was not observed on the surface of transformed sclerotic cells within 24 h after chitinase treatment (Fig 10C). Furthermore, the three-dimensional conformation of chitin distribution, which was represented by FITC-conjugated WGA, re-constructed the figuration of sclerotic cells as well as hyphal tips (Fig 11A–11D).

#### Flow cytometry assay

**Fig 12:**
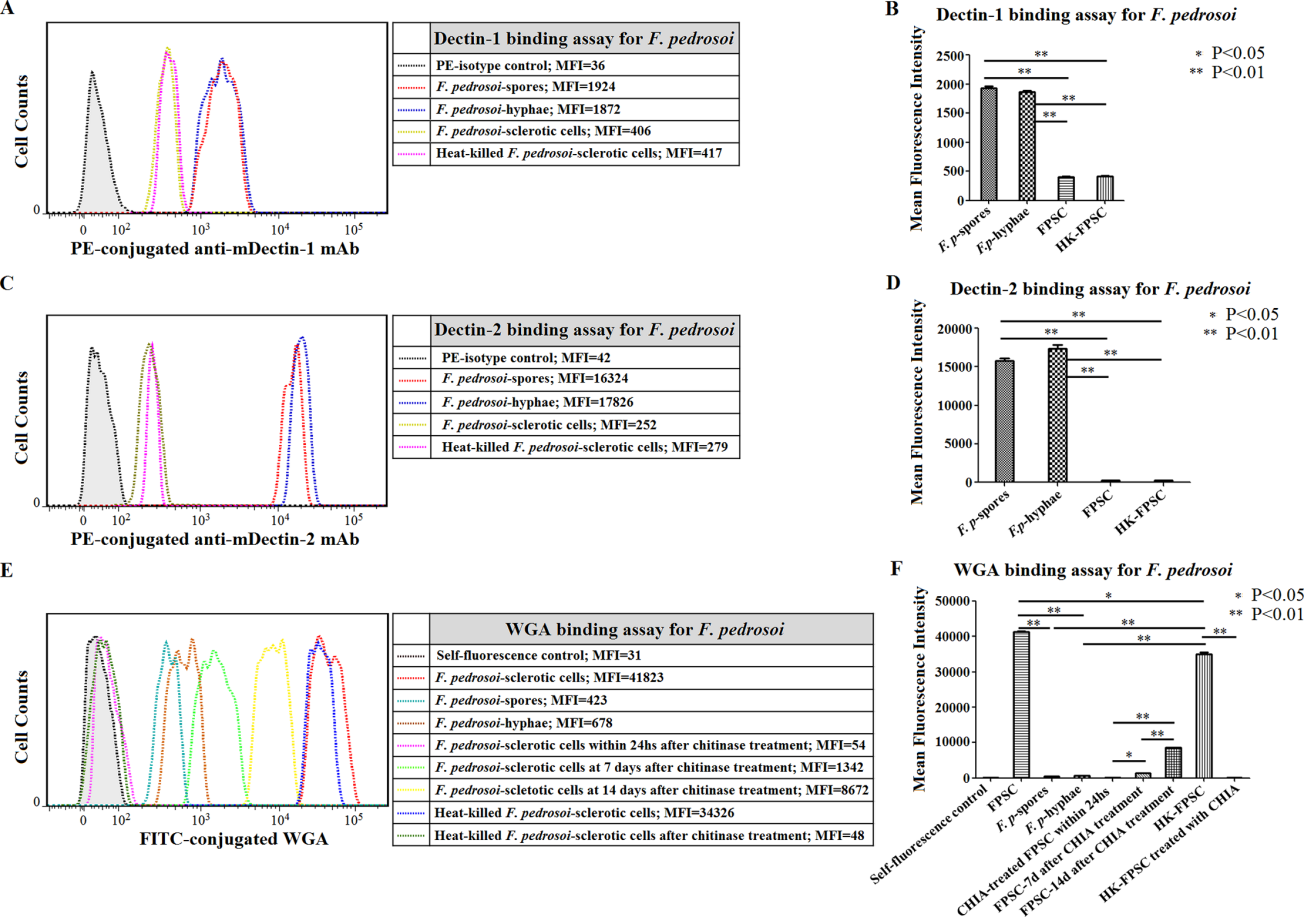
Chitin accumulation on the surface of *F*. *pedrosoi*-sclerotic cells compensatorily increased after chitinase treatment in a time-dependent manner. (A-F) Abbreviations: *F*. *pedrosoi*-spore (*F*. *p*-spore); *F*. *pedrosoi*-hyphae (*F*. *p*-hyphae); In-vitro transformed *F*. *pedrosoi*-sclerotic cells in ATCC 830 medium with 10^−6^ M PAF for 50 days (FPSC); Heat-killed FPSC (HK-FPSC); Chitinase (CHIA). (A and B) The binding capacity of murine Dectin-1 to *F*. *p-*spores, *F*. *p*-hyphae, FPSC or HK-FPSC was respectively detected by PE-conjugated anti-murine Dectin-1 mAb using flow cytometry, and was represented as mean fluorescence intensity (MFI). (C and D) The binding capacity of murine Dectin-2 to *F*. *p*-spores, *F*. *p*-hyphae, FPSC or HK-FPSC was respectively detected by PE-conjugated anti-murine Dectin-2 mAb using flow cytometry, and was represented as mean fluorescence intensity (MFI). (A-D) The fungal cells incubated only with PE-conjugated isotypes were set as blank control. (E and F) The binding capacity of FITC-conjugated WGA to *F*. *p*-spore, *F*. *p*-hyphae, FPSC or HK-FPSC before and after chitinase treatment at indicated time points was detected by flow cytometry, and was represented as mean fluorescence intensity (MFI). (B, D and F) Data represent the mean±SEM from three individual experiments performed in triplicates, and statistical analysis was performed using Univariate ANOVA and LSD-t test. Significant: ^*^ P<0.05; Highly Significant: ^**^ P<0.01.

For the group of in vitro-transformed *F*. *pedrosoi*-sclerotic cells which were cultured in ATCC 830 medium with 10^−6^ M PAF at 35°C for 50 days, the binding capacity of murine-derived Dectin-1 or Dectin-2 was significantly weaker than that for the *F*. *pedrosoi*-spores or -hyphae group (LSD-t test, p<0.01) (Fig 12A–12D). There was no significant difference in the binding capacity of Decint-1 or Dectin-2 between the *F*. *pedrosoi*-spores group and the -hyphae group (Fig 12A–12D). Meanwhile, a significant increase was detected in the binding capacity of WGA for the transformed sclerotic cells group when compared with the *F*. *pedrosoi*-spores group or -hyphae group (LSD-t test, p<0.01) (Fig 12E and 12F). There was no significant difference in the binding capacity of WGA between the *F*. *pedrosoi*-spores group and the -hyphae group (Fig 12E and 12F). Within 24 h after treatment with chitinase, the binding capacity of WGA for the transformed sclerotic cells group decreased significantly, and went down to the level of the self-fluorescence control (Fig 12E and 12F).

### Decreased accumulation of chitin on the surface of live, but not heat-killed, *F*. *pedrosoi*-sclerotic cells after chitinase treatment can be self-compensated

Within 24 h after chitinase treatment, the flow cytometry assay showed that the binding capacity of WGA to the transformed *F*. *pedrosoi*-sclerotic cells, killed by heating or not, decreased significantly than that before treatment, and went down to the level of blank control (LSD-t test, p<0.01) (Fig 12E and 12F). However, a gradual increase in the binding capacity of WGA to live, but not heat-killed, *F*. *pedrosoi*-sclerotic cells was detected at 7 days and 14 days after chitinase treatment when compared with that within 24 hours (LSD-t test, p<0.01 or p<0.05) (Fig 12E and 12F). In addition, although the binding capacity of WGA to the heat-killed sclerotic cells decreased slightly when compared with that to live cells, this binding was significantly stronger than that to *F*. *pedrosoi*-spores or -hyphae (LSD-t test, p<0.01) (Fig 12E and 12F).

### Chitin accumulation was also detected on the surface of in vivo-transformed *F*. *pedrosoi*-sclerotic cells which were isolated from the nidus with chitinase expression

**Fig 13:**
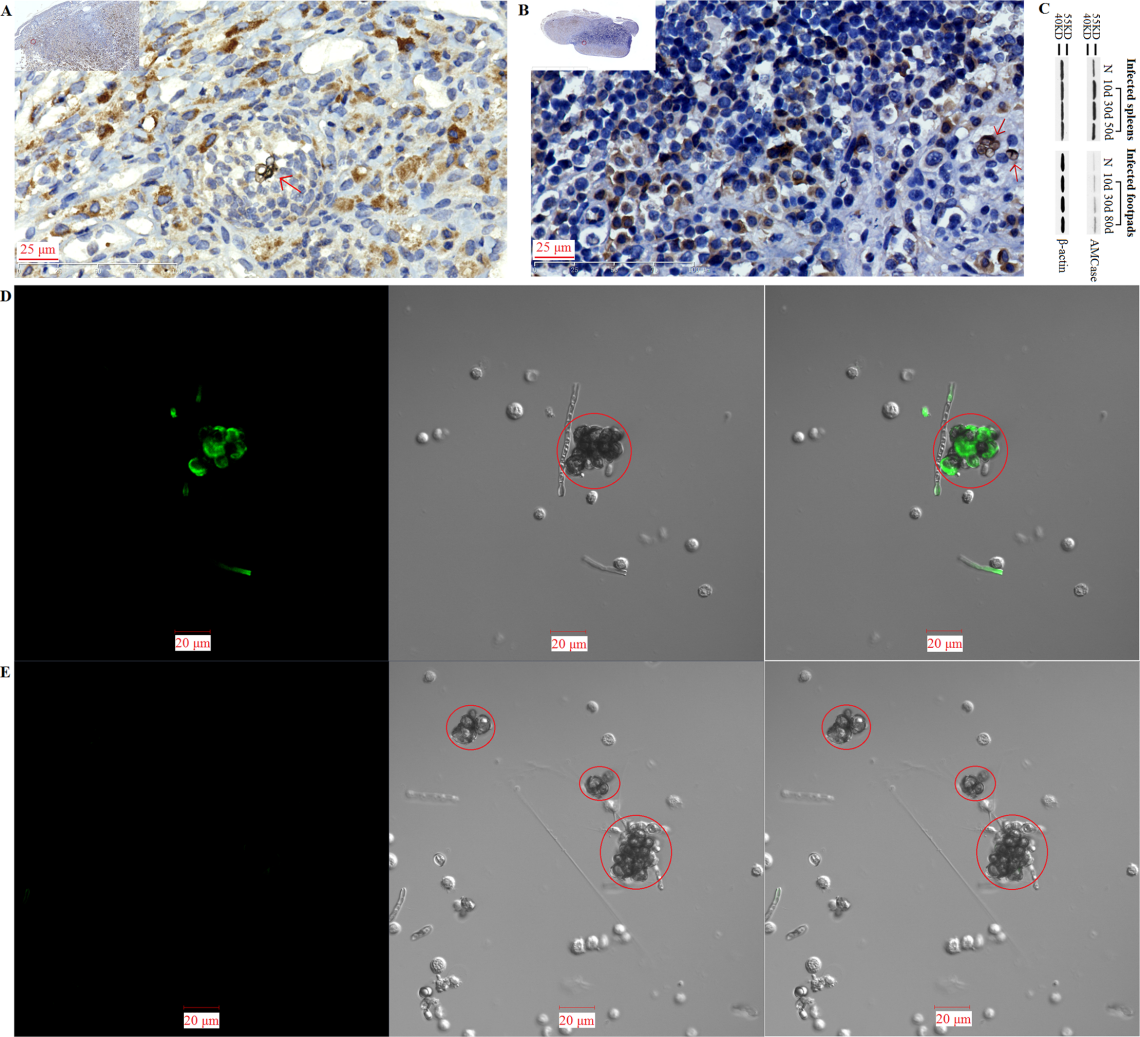
Exclusive accumulation of chitin was also detected on the surface of in-vivo transformed *F*. *pedrosoi*-sclerotic cells which were isolated from the nidus with chitinase expression. (A and B) Expression of acidic mammalian chitinase (AMCase) in the footpads of nu/nu-BALB/c mice at 80 days (A, n = 3), or in the spleens of BALB/c mice at 50 days (B, n = 3) after inoculation with *F*. *pedrosoi* was detected by rabbit anti-AMCase polyAb using immunohistochemistry. In vivo-transformed sclerotic cells in the nidus at 50 days after inoculation with *F*. *pedrosoi*-hyphae were indicated by red arrows. (C) AMCase expression in the infected footpads of nu/nu-BALB/c mice or in the infected spleens of BALB/c mice was detected by rabbit anti-AMCase polyAb using western blotting at indicated days after inoculation with *F*. *pedrosoi*-hyphae (n = 5 per subgroup of BALB/c and nu/nu- BALB/c mice). The nu/nu-BALB/c or BALB/c mice subcutaneously or intraperitoneally inoculated with NS were set as normal controls (n = 15). β-actin in the infected footpads or spleens was set as loading control, and markers were shown. (D) Confocal microscope was introduced to detect the binding of FITC-conjugated WGA to *F*. *pedrosoi* isolated from the infected spleens of BALB/c mice at 50 days post-inoculation. (E) *F*. *pedrosoi* isolated from infected spleens of BALB/c mice at 50 days post-inoculation with chitinase pretreatment was set as control. In-vivo transformed sclerotic cells were indicated by red circles. Left column: fluorescence field; Middle column: bright field; Right column: merged images. Scale bar = 20 μm.

For the *F*. *pedrosoi*-infected footpads of nu/nu-BALB/c mice at 80 days post-inoculation and spleens of BALB/c mice at 50 days post-inoculation, the agents presented themselves as sclerotic cells in tissue where obvious expression of acidic mammalian chitinase (AMCase) was detected in the cytoplasm of inflammatory cells by immunohistochemistry (Fig 13A and 13B). Furthermore, western blotting revealed that endogenous AMCase was expressed consecutively in the infected footpads and spleens at 10 days, 30 days, 50 days and 80 days after inoculation with *F*. *pedrosoi*-hyphae (Fig 13C). However, chitin accumulation was exclusively detected on the surface of transformed sclerotic cells and hyphal tips of *F*. *pedrosoi* isolated from the infected spleen with AMCase expression at 50 days post-inoculation (Fig 13D). Within 24 h after chitinase treatment, chitin distribution cannot be observed on the sclerotic cells of isolated *F*. *pedrosoi* (Fig 13E).

### Chitin contributes to an impaired IFN-γ production in the splenocytes of BALB/c mice after intraperitoneal stimulation of heat-killed *F*. *pedrosoi*-sclerotic cells

**Fig 14:**
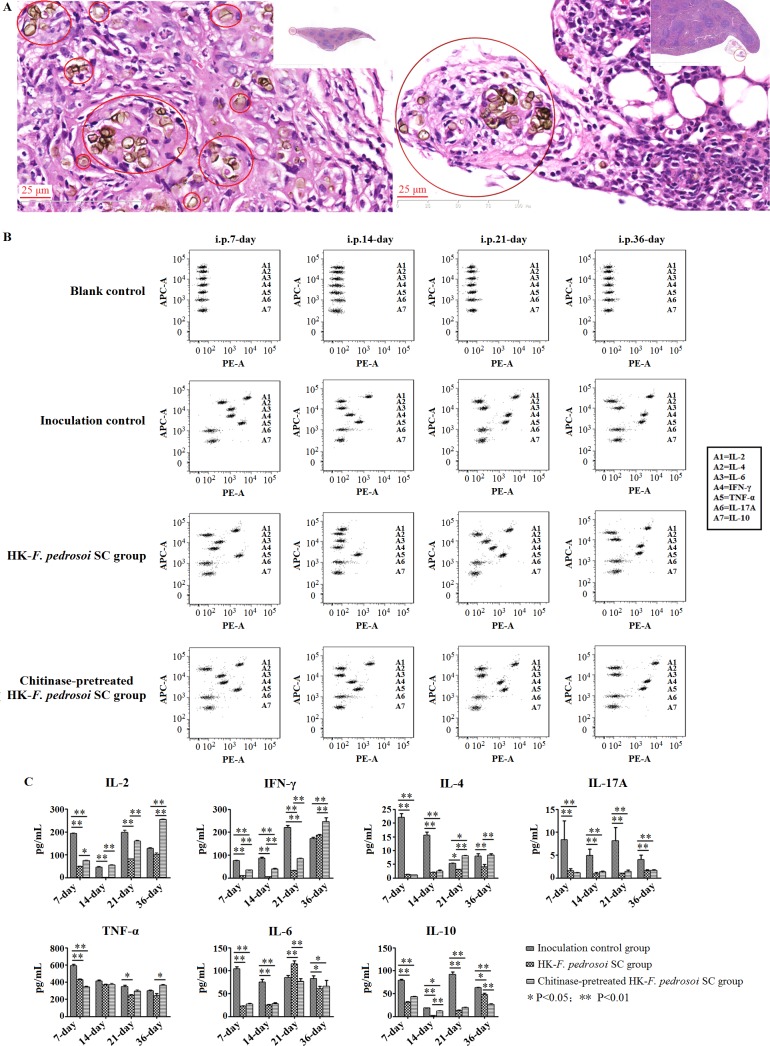
Chitin contributes to an inhibited IFN-γ production in splenocytes of BALB/c mice after intraperitoneal stimulation of heat-killed *F*. *pedrosoi* sclerotic cells. (A-C) The BALB/c mice were intraperitoneally injected for 3 times at 7-day intervals with in-vitro transformed *F*. *pedrosoi*-sclerotic cells which were heat-killed and treated with chitinase or not. (A) HE staining (×400) for the spleens isolated from BALB/c mice at 36 days after initial injection of heat-killed *F*. *pedrosoi*-sclerotic cells with chitinase treatment (left panel) or not (right panel). The sclerotic cells with cross -septation in the nidus were indicated by red circles. (B) BD Cytometric Beads Array (CBA) murine cytokine kit was introduced to measure the Th1/Th2/Th17 cytokine levels in culture supernatants of splenocytes from BALB/c mice at 7 days, 14 days, 21 days and 36 days after initial injection of heat-killed *F*. *pedrosoi*-sclerotic cells with chitinase treatment or not (n = 16 per group). The BALB/c mice intraperitoneally inoculated with normal saline were set as inoculation control (n = 16). Before measurement, the splenocytes from BALB/c mice were adjusted to 2×10^6^ cells in a volume of 2 mL RPMI1640 plus 10% FCS, and were pretreated with 1×Cell Stimulation Solution at 37°C for 6h. Seven bead populations with distinct APC-A fluorescence intensities had been coated with capture Abs specific for IL-2, IL-4, IL-6, IFN-γ, TNF-α, IL-17A and IL-10 proteins, as indicated by A1-A7 in turns. The concentration of specific cytokine mentioned above can be revealed by the fluorescence intensity of PE-conjugated detection Ab, and be calculated according to the standard curve established by cytokine standards using FCAP Array software. The assay diluent incubated with beads and PE-conjugated Ab was set as blank control. (C) Graph showing the concentration (pg/mL) of Th1/Th2/Th17 cytokines produced by splenocytes from BALB/c mice intraperitoneally stimulated with heat-killed *F*. *pedrosoi*-sclerotic cells with chitinase treatment or not as well as inoculation controls at the indicated days after initial inoculation. Data represent the mean±SEM (n = 4 per group at each indicated time point), and statistical analysis was performed using Univariate ANOVA and LSD-t test. Significant: ^*^ P<0.05; Highly Significant: ^**^ P<0.01.

After intraperitoneal injections of heat-killed *F*. *pedrosoi*-sclerotic cells in the presence or absence of chitinase treatment for 3 times at 7-day intervals, HE staining showed that the fungal inocula were largely eliminated, and the remnant sclerotic cells were well encapsulated in the spleens of BALB/c mice at 36 days after initial injection (Fig 14A).

When compared with the inoculation control group, significantly decreased levels of IFN-γ and IL-17A in the culture supernatant of splenocytes from the stimulated group in the presence or absence of chitinase treatment were consecutively detected at 7 days, 14 days and 21 days after initial inoculation (LSD-t test, p<0.01) (Fig 14B and 14C). In addition, IL-4 level in the stimulated group in the presence or absence of chitinase treatment was also significantly lower than that in the inoculation control group at 7 days and 14 days after initial inoculation (LSD-t test, p<0.01) (Fig 14B and 14C). With the elimination of fungal inocula until 36 days after initial injection, the levels of IL-2, IFN-γ as well as IL-4 in the stimulated group in the presence or absence of chitinase treatment recovered to or even surpassed those in the inoculation control group (Fig 14A–14C).

Notably, significantly increased levels of IL-2 and IFN-γ in the culture supernatant of splenocytes were consecutively detected in the stimulated group with chitinase treatment at 7 days, 14 days, 21 days and 36 days post-inoculation respectively when compared with the stimulated group without chitinase treatment (LSD-t test, p<0.05 or p<0.01) (Fig 14B and 14C). There was no significant difference in the IL-17A level between the two stimulated groups in the presence or absence of chitinase treatment during the whole 36-day observation period (Fig 14B and 14C). Furthermore, consecutive increase or decrease in the levels of pro-inflammatory cytokines, TNF-α and IL-6, as well as anti-inflammatory cytokine, IL-10, was not detected between the two groups, although some changes were observed in the levels of such cytokines during the 36-day period (Fig 14B and 14C).

## Discussion

It is well established that morphological change of *F*. *pedrosoi* to the sclerotic cell form results in an increased resistance to host immune responses in comparison with saprophytic hyphae and conidia, and as such, is linked to the survival of this agent in tissue [17, 20, 33]. In the present study, we have demonstrated that the transformation of saprophytic *F*. *pedrosoi* into sclerotic cells facilitates the chronic development of chromoblastomycosis in BALB/c mice by intraperitoneal injection of *F*. *pedrosoi*-spores, -hyphae and in-vitro induced sclerotic cells.

It is known that human neutrophils can effectively kill pathogenic fungi in a ROS-dependent manner, and thus play a pivotal role in host defense against invasive fungal infection [36, 37]. Using an experimental model of murine chromoblastomycosis due to *F*. *pedrosoi*, a recent study has also pointed out that the sclerotic cells are absent or destroyed in the neutrophilic center of abscess but not in foamy macrophage-rich region of infected tissue [20]. In addition, the neutrophils were also reported to have fungicidal action in vitro and limited phagocytic ability against *F*. *pedrosoi* [38]. Systemically, however, T lymphocytes contribute greatly to the control of this disease, as shown in our previous study [8]. Here we showed that although neutrophil infiltration was obvious in the infected spleen of BALB/c mice at 10 days after intraperitoneal inoculation with induced sclerotic cells, an impaired ROS generation by neutrophils simultaneously with decreased level of INF-γ was observed in comparison with the groups intraperitoneally inoculated with *F*. *pedrosoi*-spores or -hyphae. Considering that Th1-produced INF-γ optimally promotes ROS generation in neutrophils and activates subsequent fungicidal processes [9, 10, 39], we infer that the transformation of *F*. *pedrosoi* into sclerotic cells will attenuate neutrophil ROS-dependent killing of this agent in tissue, and therefore contribute to the refractoriness of experimental chromoblastomycosis in mice. On this basis, we have further demonstrated that intraperitoneal administration of rmIFN-γ greatly reduces the fungal burden in the spleens of BALB/c mice infected with *F*. *pedrosoi*-sclerotic cells and inhibits peritoneal dissemination of this agent. In addition, we have also shown that exogeneous rmIFN-γ contributes to the formation and maintenance of micro-abscess, and restores the decrease in neutrophil ROS generation in the spleen infected with *F*. *pedrosoi*-sclerotic cells during the whole observation period. By contrast, for the groups intraperitoneally inoculated with *F*. *pedrosoi*-hyphae or sclerotic cells, an inhibited IL-17A production in the spleen can be only detected in the latter phase of infection. Meanwhile, increased levels of IFN-γ and IL-2, but not IL-17A, were observed in the spleen of BALB/c mice until elimination of the inoculated *F*. *pedrosoi*-spores. Accordingly, although an inhibited IL-17A production during the later stage of infection may attenuate neutrophil infiltration in the nidus, we believe that the decreased level of IFN-γ during the whole period of infection will persistently impair neutrophil ROS-mediated killing of *F*. *pedrosoi*-sclerotic cells. These findings, to some extent, suggest a causal link between a decreased level of IFN-γ and the refractoriness of experimental chromoblastomycosis in mice due to *F*. *pedrosoi*, which is corroborated by recent clinical observations [14–16]. What’s more, we assume that the sclerotic cells have some unknown characteristics which may be involved in the impairment of original Th1/Th2/Th17 cytokine patterns in mice.

Notably, although the production of Th1/Th2/Th17 cytokines was substantially inhibited in the BALB/c mice intraperitoneally infected with live *F*. *pedrosoi*-sclerotic cells in comparison with the inoculation control group until 30 days post inoculation, an increased secretion of TNF-α, an important pro-inflammatory mediator, was simultaneously observed in this infected group. Combined with the clinical and histopathological features in BALB/c mice intraperitoneally infected with *F*. *pedrosoi*-sclerotic cells, we infer that the intensity of inflammation in chromoblastomycosis cannot well reflect the status of host-protective immune responses. It is the Th cell development that, to a large extent, predicts the outcome of this disease, consistent with the findings published previously [14–16].

It is well established that most of fungal chitin microfibriles are located immediately adjacent to the plasma membrane in the lateral cell wall [26, 29]. Mannoproteins are bound to β-1,6-glucan, which in turn is attached to the reducing end of β-1,3-glucan, and this configuration allows cell wall remodeling and growth [23, 26, 29]. However, at the septum of mother-daughter neck, chitin bound to β-1, 3-glucan competes out the β-1, 6-glucan and the mannoproteins attached to it, and thus form the chitin ring [28, 29]. This change in chemical linkages greatly increases chitin exposure on the septum [25, 29]. Interestingly, recent studies in *F*. *pedrosoi* have suggested that it is the characteristic multi-septations in the mother sclerotic cell that constitute the cell wall of daughter sclerotic cell [40, 41]. In the present study, we further demonstrate that some chitin-like component exclusively accumulates on the surface and multi-septated planes of sclerotic cells induced in vitro or isolated from tissue, but not on the saprophytic spores and hyphae of *F*. *pedrosoi*. Thus, our observations indicate that there may be changes in chemical linkages between chitin and other parts on the cell wall during the transformation of this agent into parasitic sclerotic cell from. Recent studies have reported that chitin acts as a size- and concentration-dependent stimulus of IL-10 and TNF in a manner that involves MR, TLR2 and Dectin-1 [42, 43]. However, we observed that the binding of murine-derived Dectin-1, MR and Dectin-2 were mainly restricted to the cell surface of *F*. *pedrosoi* hyphae and spores, but not the transformed sclerotic cells. In addition, we further demonstrate that chitin accumulation on the cell wall of transformed sclerotic cells has no obvious effect on the secretion of TNF-α and IL-10 upon in vivo stimulation. This indicates that the chitin component on sclerotic cells of *F*. *pedrosoi* may have different immunological characteristics, and there may be some other unknown PRRs which are responsible for recognition of it.

It should be mentioned that our in vitro experiments had previously demonstrated that the chitin-like component on sclerotic cells of *F*. *pedrosoi* were able to inhibit Dectin-1-mediated murine Th17 development by masking β-glucans [8]. In the present study, we primarily demonstrate that it is the chitin-like component that contributes to an impaired IFN-γ and IL-2 production in splenocytes from BALB/c mice intraperitoneally injected with heat-killed *F*. *pedrosoi* sclerotic cells. However, decreased production of IL-17A in splenocytes from BALB/c mice intraperitoneally infected with sclerotic cells is irrelevant to chitin component. It should be noted that we observed effective phagocytosis of sclerotic cells by macrophages in the infected murine footpads and spleens, but not by in vitro cultured splenocytes. Therefore, we inferred that the chitin recognition on sclerotic cells by different PRRs and subsequent signaling pathways triggered by them may be involved in above-mentioned difference in murine Th cytokine profiles.

The discovery of human chitinases that are constitutively expressed by macrophages and epithelial cells suggests the presence of a first line of defense against chitin-containing pathogens, as well as mechanisms for chitin recognition, breakdown and immune-modulation in the human host [43, 44]. In this study, we also demonstrate that the intensity of chitin component on in vitro-induced sclerotic cells of *F*. *pedrosoi* can be gradually recovered in a time-dependent manner after chitinase treatment. Although endogenous expression of chitinase was detected consecutively in the infected mice footpads and spleens, we observed exclusive accumulation of chitin on the cell wall of sclerotic cells which were isolated from BALB/c mice intraperitoneally infected with *F*. *pedrosoi*. Accordingly, we inferred that the transformed sclerotic cells of *F*. *pedrosoi* have the potential to maintain the integrity of chitin on the outer cell wall in a manner dependent on self-renewing and compensatory synthesis when confronted with chitinase produced by host immune cells.

Therefore, the findings in the present study partly corroborate our hypothesis that the transformation of *F*. *pedrosoi* into sclerotic cell form can impair the original immunocompetent Th cell cytokine pattern in BALB/c mice, especially IFN-γ production, via a mechanism involving the “chitin accumulation effect” mentioned above.

In addition, considering that chitin digestion cannot completely eliminate the inhibitory effect of sclerotic cells on IFN-γ production as demonstrated in the present study, we believe that some mechanisms other than “chitin accumulation effect” may also contribute to the inhibition of murine Th1 development with the transformation of saprophytic *F*. *pedrosoi* into parasitic sclerotic cells.

In sum, this study extends our understanding of the linkage between transformation of saprophytic *F*. *pedrosoi* into sclerotic cell form and the refractoriness of chromoblastomycosis due to *F*. *pedrosoi*.
